# Supplementary material for: Classification of road traffic injury collision characteristics using text mining analysis: Implications for road injury prevention
Source: PLoS One. 2021 Jan 27;16(1):e0245636. doi: 10.1371/journal.pone.0245636 (PMC7840051; doi:10.1371/journal.pone.0245636)
Supplement: S1 File — (DOCX) [file pone.0245636.s001.docx]

# S1 File: Exclusion dictionary

4_WHEEL_DRIVE

A

ABLE

ABOUT

ABOVE

ACCIDENT_AS_PER_AMBULANCE_REPORT

ACCORDING

ACCORDINGLY

ACTUALLY

ADVISES

AFTER

AFTERWARDS

AGAIN

AGAINST

AINT

ALL

ALLOW

ALLOWS

ALMOST

ALONE

ALONG

ALREADY

ALSO

ALTHOUGH

ALWAYS

AM

AMBULANCE_REPORT

AMONG

AMONGST

AN

AND

ANY

ANYHOW

ANYWAY

ANYWAYS

ANYWHERE

APART

APPRECIATE

APPROPRIATE

ARE

ARENT

AS

AS_PER_AMBULANCE

ASIDE

ASK

ASKING

AT

AV_REPORT

AVAILABLE

AWAY

B

BE

BECAME

BECOME

BECOMES

BECOMING

BEEN

BEFOREHAND

BEING

BELIEVE

BELOW

BESIDE

BESIDES

BEST

BETTER

BETWEEN

BEYOND

BOTH

BOUND

BRIEF

BUT

BY

C

CAN

CANNOT

CANT

CANT

CANT_REMEMBER

CAUSE

CAUSES

CERTAIN

CERTAINLY

CHANGES

CIRCUMSTANCES_NOT_KNOWN

CLAIM_FORM

CLAIM_LODGEMENT

CLEARLY

CMON

CO

COM

COME

COMES

CONCERNING

CONSIDER

CONSIDERING

CONTAIN

CONTAINING

CONTAINS

CORRESPONDING

COULD

COULDNT

COURSE

CS

CURRENTLY

D

DEFINITELY

DESCRIBED

DESCRIPTION

DESPITE

DIAGRAM

DIFFERENT

DO

DOES

DOESNT

DOING

DONE

DONT

DOWN

DOWNWARDS

DUE

DURING

E

EACH

EAST

EASTERN

EASTERN_FREEWAY

ED_NOTES

EDU

EG

EIGHT

EITHER

ELSE

ELSEWHERE

ENOUGH

ENTIRELY

ESPECIALLY

ET

ETC

EVEN

EVER

EVERY

EVERYWHERE

EX

EXACTLY

EXAMPLE

EXCEPT

F

FAR

FEW

FIFTH

FIND

FIRST

FIVE

FOLLOWED

FOLLOWING

FOLLOWS

FOR

FORMER

FORMERLY

FORTH

FOUND

FOUR

FOUR_WHEEL_DRIVE

FROM

FURTHER

FURTHERMORE

G

GET

GETS

GETTING

GIVE

GIVEN

GIVES

GO

GOES

GOING

GONE

GOT

GOTTEN

GREETINGS

H

HAD

HADNT

HAPPENS

HARDLY

HAS

HASNT

HAVE

HAVENT

HAVING

HE

HELLO

HELP

HENCE

HER

HERE

HEREAFTER

HEREBY

HEREIN

HERES

HEREUPON

HERS

HERSELF

HES

HI

HIM

HIMSELF

HIS

HITHER

HOME

HOPEFULLY

HOSPITAL

HOUSE

HOW

HOWBEIT

HOWEVER

HUME_HIGHWAY

I

ID

IE

IF

IGNORED

ILL

IM

IMMEDIATE

INASMUCH

INC

INCIDENT_NUMBER

INDEED

INFORMATION

INNER

INSOFAR

INSTEAD

INTO

INVOLVED_IN

INWARD

IS

ISNT

IT

ITD

ITLL

ITS

ITS

ITSELF

IVE

J

JUST

K

KEEP

KEEPS

KEPT

KM

KNOW

KNOWN

KNOWS

L

LATELY

LATER

LATTER

LATTERLY

LEAST

LESS

LEST

LET

LETS

LIKE

LIKED

LIKELY

LITTLE

LOOK

LOOKING

LOOKS

LTD

M

MADE

MAINLY

MAKE

MANY

MAY

MAYBE

MEAN

MEANWHILE

MERELY

METER

METRE

MIGHT

MINE

MORE

MOREOVER

MOST

MOSTLY

MOTOR

MUCH

MUST

MY

MYSELF

N

NAME

NAMELY

ND

NEAR

NEARLY

NECESSARY

NEED

NEEDS

NEITHER

NEVER

NEVERTHELESS

NEW

NEXT

NINE

NO

NOR

NORMALLY

NORTH

NORTHERN

NOT

NOT_LISTED

NOT_MEDICALLY_ABLE

NOTES_PATIENT

NOVEL

NOW

O

OBVIOUSLY

OF

OFF

OFTEN

OH

OLD

ON

ONCE

ONE

ONES

ONLY

ONTO

OR

OTHER

OTHERS

OTHERWISE

OUGHT

OUR

OURS

OURSELVES

OUT

OUTSIDE

OVER_XXXX_ROAD

OVERALL

OWN

P

PARTICULAR

PARTICULARLY

PER

PERHAPS

PLACED

PLEASE

PLUS

POLICE_INVESTIGATION

POLICE_REPORT

POSSIBLE

PRESUMABLY

PROBABLY

PROVIDES

PUT

Q

QUE

QUITE

QV

R

RATHER

RE

REALLY

REASONABLY

REEFER_TO_POLICE

REF_POLICE_REPORT

REFER

REGARDING

REGARDLESS

REGARDS

RELATIVELY

RESPECTIVELY

S

SAID

SAME

SAY

SAYING

SAYS

SECOND

SECONDLY

SEE_POLICE_REPORT

SEEM

SEEMED

SEEMING

SEEMS

SEEN

SELF

SELVES

SENSIBLE

SENT

SERIOUS

SERIOUSLY

SEVEN

SEVERAL

SHALL

SHE

SHOULD

SHOULDNT

SINCE

SIX

SO

SOME

SOMEHOW

SOMETHING

SOMETIME

SOMETIMES

SOMEWHAT

SOON

SORRY

SOUTH

SOUTHERN

SPECIFIED

SPECIFY

SPECIFYING

START

STATES

SUB

SUCH

SUP

SURE

T

TAKE

TAKEN

TBA

TBC

TELL

TENDS

TH

THAN

THANK

THANKS

THANX

THAT

THATS

THATS

THE

THEIR

THEIRS

THEM

THEMSELVES

THEN

THENCE

THERE

THEREAFTER

THEREBY

THEREFORE

THEREIN

THERES

THERES

THEREUPON

THESE

THEY

THEYD

THEYLL

THEYRE

THEYVE

THINK

THIRD

THIS

THOROUGH

THOROUGHLY

THOSE

THOUGH

THREE

THROUGHOUT

THUS

TO

TOGETHER

TOO

TOOK

TRIED

TRIES

TRULY

TRY

TRYING

TS

TWICE

TWO

U

UN

UNABLE_TO_DESCRIBE

UNABLE_TO_RECALL

UNDER

UNFORTUNATELY

UNKNOWN

UNLESS

UNLIKELY

UNTIL

UNTO

UP

UPON

US

USE

USED

USEFUL

USES

USING

USUALLY

UUCP

V

VALLEY_HIGHWAY

VALUE

VARIOUS

VERY

VIA

VIZ

VS

W

WANT

WANTS

WAS

WASNT

WAY

WE

WED

WELCOME

WELL

WELL

WENT

WERE

WERENT

WEST

WESTERN

WEVE

WHAT

WHATEVER

WHATS

WHEN

WHENCE

WHENEVER

WHERE

WHEREAFTER

WHEREAS

WHEREBY

WHEREIN

WHERES

WHEREUPON

WHEREVER

WHETHER

WHICH

WHILE

WHILST

WHITHER

WHO

WHOEVER

WHOLE

WHOM

WHOS

WHOSE

WHY

WILL

WILLING

WISH

WITH

WITHIN

WITHOUT

WONDER

WONT

WORK

WOULD

WOULD

WOULDNT

X

XXXX

Y

YES

YET

YOU

YOUD

YOULL

YOUR

YOURE

YOURS

YOURSELF

YOURSELVES

YOUVE

Z

ZERO
