# Supplementary material for: Classification of road traffic injury collision characteristics using text mining analysis: Implications for road injury prevention
Source: PLoS One. 2021 Jan 27;16(1):e0245636. doi: 10.1371/journal.pone.0245636 (PMC7840051; doi:10.1371/journal.pone.0245636)
Supplement: S2 File — (DOCX) [file pone.0245636.s002.docx]

S2 File: Lemmatization dictionary

4WD=MOTOR_VEHICLE

ABANDONED=ABANDON

ABANDONING=ABANDON

ABRUPTLY=SUDDENLY

ABSORBED=ABSORB

ABSORBING=ABSORB

ABUSED=ABUSE

ABUSES=ABUSE

ACCELARATOR=ACCELERATOR

ACCELERATED=ACCELERATOR

ACCELERATING=ACCELERATOR

ACCELERATOR=ACCELERATOR

ACCEPTED=ACCEPT

ACCESSING=ACCESS

ACCIDENT=ACCIDENT

ACCUSED=ACCUSE

ACCUSES=ACCUSE

ACQUIRED=ACQUIRE

ACQUIRING=ACQUIRE

ADAPTED=ADAPT

ADDED=AD

ADDING=AD

ADDRESSING=ADDRESS

ADMIRED=ADMIRE

ADMIRING=ADMIRE

ADOPTED=ADOPT

AGREED=AGREE

AIDED=AID

AIDING=AID

AIMED=AIM

AIMING=AIM

AIRBAGS=AIRBAG

AIRHORN=WARN

ALANS=ALAN

ALCOHOL=SUBSTANCES

ALIGHT=FIRE

ALLEGED=ALLEGE

ALLEGING=ALLEGE

ALONGSIDE=ALONG

ALRIGHT=OK

AM=BE

AMBER=AMBERLIGHT

AMERICANS=AMERICAN

AMERICAS=AMERICA

ANALYSED=ANALYSE

ANALYSES=ANALYSIS

ANALYSING=ANALYSE

ANIMALS=ANIMAL

ANNS=ANN

APOLOGISED=APOLOGISE

APOLOGISES=APOLOGISE

APOLOGISING=APOLOGISE

APPARENTLY=ALLEGEDLY

APPEARED=APPEAR

APPEARS=APPEAR

APPENDICES=APPENDIX

APPROACHED=APPROACH

APPROACHING=APPROACH

APPROX=APPROXIMATELY

AQUAPLANE=IMPACT_MOTION

aquaplaned=aquaplane

ARE=BE

ARISEN=ARISE

AROSE=ARISE

AROUSED=AROUSE

AROUSES=AROUSE

ARRANGED=ARRANGE

ARRANGING=ARRANGE

ARTHURS=ARTHUR

ASHLEYS=ASHLEY

ASKING=ASK

ASSAULTED=INTENTIONAL

ASSESSING=ASSESS

ASSURED=ASSURE

ATE=EAT

ATTAINED=ATTAIN

ATTEMPTED=ATTEMPT

ATTEMPTING=ATTEMPT

ATTRIBUTED=ATTRIBUTE

AUDI=MOTOR_VEHICLE

AVE=ROADWAY

AVOIDED=AVOID

AVOIDING=AVOID

AWAITED=AWAIT

AWAITING=AWAIT

AWOKE=FATIGUE

AXES=AXIS

BACKED=BACKWARD

BACKED=REVERSE

BACKING=BACKWARD

BACKING=REVERSE

BACKWARDS=BACKWARD

BACTERIA=BACTERIUM

BADE=BID

BANGED=BANG

BANGING=BANG

BAREST=BARE

BARRIERS=BARRIER

BASE=BASE

BASES=BASE

BASS=BASS

BEACH=SOIL

BEARING=BEAR

BEATEN=BEAT

BEATING=BEAT

BECAME=BECOME

BECOMING=BECOME

BEEN=BE

BEGAN=BEGIN

BEGUN=BEGIN

BEHAVIOURS=BEHAVIOUR

BEHIND=REAR

BEING=BE

BELONGED=BELONG

BELONGING=BELONG

BENDS=BEND

BENEFITED=BENEFIT

BENEFITING=BENEFIT

BENEFITTED=BENEFIT

BENEFITTING=BENEFIT

BENT=BEND

BEST=GOOD

BETTER=GOOD

BICYCLE=PEDAL_BIKE

BICYCLE=PEDAL_BIKE

BICYCLIST=CYCLIST

BIDDEN=BID

BIGGER=BIG

BIGGEST=BIG

BIKES=BIKE

BILE=BIKE

BILES=BILE

BIND=BOUND

BIRD=ANIMAL

BITING=BITE

BITUMEN=ROAD_SURFACE

BLACK=DARK

BLACKED=BLACKOUT

BLACKER=BLACK

BLAMED=BLAME

BLAMING=BLAME

BLESSING=BLESS

BLINDER=BLIND

BLINKER=INDICATE

BLINKERS=INDICATE

BLOWED=BLOW

BLOWN=BLOW

BLVD=ROADWAY

BMW=MOTOR_VEHICLE

BOLDER=ROCK

BOLDEST=BOLD

BOLLARDS=BARRIER

BONNED=TBONE

BONNETT=BONNET

BONUSES=BONUS

BOOKED=BOOK

BORE=BEAR

BORNE=BEAR

BORROWINGS=BORROWING

BOSS=BOSS

BOUGHT=BUY

BOULDER=ROCK

BOUND=BIND

BOUNDED=BOUND

BOYFRIEND=PARTNER

BOYFRIENDS=PARTNER

BRADFORDS=BRADFORD

BRAKED=BRAKE

BRAKES=BRAKE

BRAKING=BRAKE

BRANCH=TREE

BRAVER=BRAVE

BRAVEST=BRAVE

BRAZILS=BRAZIL

BREAKING=BREAK

BREATHED=BREATHE

BRED=BREED

BRETHREN=BROTHER

BRIDGES=BRIDGE

BRIEFER=BRIEF

BRIEFEST=BRIEF

BRIGHTER=BRIGHT

BRIGHTEST=BRIGHT

BRINGING=BRING

BROADER=BROAD

BROADEST=BROAD

BROKE=BREAK

BROKEN=BREAK

BROTHER=SIBLING

BROTHERS=SIBLING

BROUGHT=BRING

BROWNER=BROWN

BRUCES=BRUCE

BRUSHED=CLIPPED

BUGGY=SCOOTER

BUILDING=BUILDING

BUILT=BUILD

BULLBAR=BUMPER

BUREAUS=BUREAU

BUREAUX=BUREAU

BURNED=BURN

BURNT=BURN

BURST=BURST

BUSES=BUS

BUSH=TREE

BUSHES=TREE

BUSINESSMEN=BUSINESSMAN

CALLED=CALL

CALLING=CALL

CALMED=CALM

CALMER=CALM

CALMING=CALM

CALVES=CALF

CAME=COME

CAMPBELLS=CAMPBELL

CAMRY=MOTOR_VEHICLE

CANCELLED=CANCEL

CANCELLING=CANCEL

CANVASES=CANVAS

CAPTURED=CAPTURE

CAR=MOTOR_VEHICLE

CARAVAN=TOWING

CARED=CARE

CAREERED=VEER

CARERS=CARER

CARING=CARE

CARRIAGEWAY=LANE

CARRIER=CARRIER

CARS=MOTOR_VEHICLE

CARTWHEELED=AIRBORNE

CASE=CASE

CASES=CASE

CASH=CASH

CASING=CAUSE

CAST=CAST

CAT=ANIMAL

CATAPULTED=AIRBORNE

CATHOLICS=CATHOLIC

CAUGHT=CATCH

CAUSED=CAUSE

CAUSED=CAUSE

CAUSES=CAUSE

CAUSING=CAUSE

CDS=CD

CEASES=CEASE

CENSUSES=CENSUS

CENTER=CENTRE

CENTRED=CENTRE

CENTRES=CENTRE

CENTRING=CENTRE

CFA=FIRST_RESPONDERS

CHAIRED=CHAIR

CHAIRING=CHAIR

CHAIRMEN=CHAIRMAN

CHALLENGED=CHALLENGE

CHALLENGING=CHALLENGE

CHANGED=CHANGE

CHANGING=CHANGE

CHAPMANS=CHAPMAN

CHARACTERISED=CHARACTERISE

CHARACTERISES=CHARACTERISE

CHARACTERISING=CHARACTERISE

CHARACTERISTICS=CHARACTERISTIC

CHASE=PURSUE

CHASED=PURSUE

CHASES=CHASE

CHASING=PURSUE

CHEAPER=CHEAP

CHEAPEST=CHEAP

CHECKED=CHECK

CHECKING=CHECK

CHEESES=CHEESE

CHEQUES=CHEQUE

CHESHIRES=CHESHIRE

CHILDREN=CHILD

CHORUSES=CHORUS

CHOSE=CHOOSE

CHOSE=DECIDE

CHOSEN=CHOOSE

CHRISTIANS=CHRISTIAN

CHRISTS=CHRIST

CITED=CITE

CITING=CITE

CLAIMANT=CLIENT

CLAIMED=CLAIM

CLAIMING=CLAIM

CLARKES=CLARKE

CLASSICS=CLASSIC

CLAUSES=CLAUSE

CLEANED=CLEAN

CLEANEST=CLEAN

CLEANING=CLEAN

CLEARED=CLEAR

CLEARER=CLEAR

CLEAREST=CLEAR

CLEVERER=CLEVER

CLEVEREST=CLEVER

CLIENTS=CLIENT

CLIMBED=CLIMB

CLINGING=CLING

CLINICS=CLINIC

CLINTONS=CLINTON

CLIPPED=CLIPPED

CLIPPING=CLIPPED

CLOSER=CLOSE

CLUNG=CLING

COINCIDED=COINCIDE

COLDER=COLD

COLDEST=COLD

COLLAPSED=COLLAPSE

COLLEAGUES=WORK

COLLECTED=CLIPPED

COLLIDE=COLLIDE

COLLIDED=COLLIDE

COLLIDING=COLLIDE

COLLISION=COLLISION

COLLISIONS=COLLISION

COLLISON=COLLISION

COLOURED=COLOUR

COLOURING=COLOUR

COLOURS=COLOUR

COMBINED=COMBINE

COMBINING=COMBINE

COMETH=COME

COMIN=COME

COMING=COME

COMMANDED=COMMAND

COMMENCED=START

COMMENCING=START

COMMODORE=MOTOR_VEHICLE

COMPARED=COMPARE

COMPARING=COMPARE

COMPELLING=COMPEL

COMPETED=COMPETE

COMPETENCES=COMPETENCE

COMPETING=COMPETE

COMPILED=COMPILE

COMPILING=COMPILE

COMPLAINED=COMPLAIN

COMPLETED=COMPLETE

COMPLETING=COMPLETE

CONCEDED=CONCEDE

CONCEDING=CONCEDE

CONCENTRATING=CONCENTRATION

CONCERNED=CONCERN

CONCLUDED=CONCLUDE

CONCLUDING=CONCLUDE

CONDEMNED=CONDEMN

CONDEMNING=CONDEMN

CONFERRING=CONFER

CONFESSING=CONFESS

CONFINED=CONFINE

CONFINING=CONFINE

CONFUSED=CONFUSE

CONFUSES=CONFUSE

CONSCIOUS=BLACKOUT

CONSCIOUSNESS=BLACKOUT

CONSCIOUSNESSES=CONSCIOUSNESS

CONSEQUENCE=RESULT

CONSTITUTED=CONSTITUTE

CONSTRAINT=CONSTRAINT

CONTAINED=CONTAIN

CONTEMPTS=CONTEMPT

CONTINUATION=CONTINUE

CONTINUED=CONTINUE

CONTINUING=CONTINUE

CONTRIBUTED=CONTRIBUTE

CONTROLLED=CONTROL

CONTROLLING=CONTROL

CONVEYED=CONVEY

CONVOY=GROUP

CONVULSING=MEDICAL_CONDITION

COOKED=COOK

COOKING=COOK

COOLED=COOL

COOLER=COOL

COOLEST=COOL

COOLING=COOL

COPED=COPE

COPING=COPE

CORBETTS=CORBETT

CORRECTED=CORRECT

CORRECTING=CORRECT

CORRECTLY=CORRECT

CORRESPONDED=CORRESPOND

CORRUGATIONS=BUMP

COST=COST

COUNCILLORS=COUNCILLOR

COUSIN=FAMILY_OTHER

COUSINS=FAMILY_OTHER

COVERAGES=COVERAGE

COW=ANIMAL

CRASH=CRASH

CRASHED=CRASH

CREDITED=CREDIT

CREDITING=CREDIT

CREEPING=CREEP

CREPT=CREEP

CRESCENT=ROADWAY

CREST=RIDGE

CRESTED=RIDGE

CRESTS=RIDGE

CRISES=CRISIS

CRITERIA=CRITERION

CRITICS=CRITIC

CROSSED=CROSS

CROSSING=CROSS

CROSSROAD=INTERSECTION

CROSSROADS=INTERSECTION

CRUDER=CRUDE

CRUDEST=CRUDE

CRUELLER=CRUEL

CRUELLEST=CRUEL

CRUMPLING=CRASH

CRUSH=CRASH

CRUSHED=CRASH

CULVERT=EMBANKMENT

CURED=CURE

CURING=CURE

CURIOUSER=CURIOUS

CURLING=CURL

CURRICULA=CURRICULUM

CURVED=CURVE

CURVING=CURVE

CURVY=CURVE

CUTTING=CUT

CYLIST=CYCLIST

DAD=PARENT

DADS=PARENT

DAMPER=DAMP

DARED=DARE

DARING=DARE

DARKER=DARK

DATABASES=DATABASE

DATUM=DATA

DAUGHTER=OFFSPRING

DAUGHTERS=OFFSPRING

DAVIDS=DAVID

DAWN=VISIBILITY

DEAD=DIED

DEALING=DEAL

DEALT=DEAL

DEARER=DEAR

DEAREST=DEAR

DEBRIS=SOIL

DECEASED=DIED

DECIDED=DECIDE

DECIDED=DECIDE

DECLARED=DECLARE

DECLARING=DECLARE

DECLINED=DECLINE

DECREASES=DECREASE

DEEMED=DEEM

DEEMING=DEEM

DEEPER=DEEP

DEEPEST=DEEP

DEFEATED=DEFEAT

DEFEATING=DEFEAT

DEFENCES=DEFENCE

DEFINED=DEFINE

DEFINING=DEFINE

DEG=DEGREE

DEGREES=DEGREE

DEMANDED=DEMAND

DENYS=DENY

DEPARTED=DEPART

DEPOSITED=DEPOSIT

DEPOSITING=DEPOSIT

DEPRESSION=EMBANKMENT

DERBYS=DERBY

DESIRED=DESIRE

DESIRING=DESIRE

DESKTOPS=DESKTOP

DESTROYED=DESTROY

DETERMINED=DETERMINE

DETERMINING=DETERMINE

DEVELOPED=DEVELOP

DEVELOPING=DEVELOP

DEVONS=DEVON

DEVOTED=DEVOTE

DEVOTING=DEVOTE

DID=DO

DIED=DIE

DIES=DIE

DIRECTIONS=DIRECTION

DISAGREED=DISAGREE

DISCS=DISC

DISCUSSING=DISCUSS

DISEASES=DISEASE

DISLIKED=DISLIKE

DISLIKING=DISLIKE

DISMISSING=DISMISS

DISMOUNTED=DISMOUNT

DISMOUNTING=DISMOUNT

DISTRIBUTED=DISTRIBUTE

DISTURBED=DISTURB

DISTURBING=DISTURB

DITCH=EMBANKMENT

DIVIDED=DIVIDE

DIVIDER=BARRIER

DIVIDERS=BARRIER

DIZZY=MEDICAL_CONDITION

DNAS=DNA

DODGE=EVASIVE

DOG=ANIMAL

DOING=DO

DONE=DO

DOTH=DO

DOUBTED=DOUBT

DOUBTING=DOUBT

DR=ROADWAY

DRAGGING=DRAGGED

DRAIN=EMBANKMENT

DRAINED=DRAIN

DRANK=DRINK

DRAWING=DRAW

DRAWN=DRAW

DREAMT=DREAM

DREW=DRAW

DRIFT=VEER

DRIFTED=VEER

DRILLED=DRILL

DRILLING=DRILL

DRIVEN=DRIVE

DRIVERS=DRIVER

DROVE=DRIVE

DRUG=SUBSTANCES

DRUNK=SUBSTANCES

DRUNKER=DRUNK

DUG=DIG

DULLER=DULL

DULLEST=DULL

DUNCANS=DUNCAN

DUNE=SOIL

DUST=VISIBILITY

DUSTY=VISIBILITY

DYING=DIE

EARNED=EARN

EARTH=SOIL

EASES=EASE

EATEN=EAT

EATING=EAT

ECHIDNA=ANIMAL

ECHOED=ECHO

ECHOES=ECHO

ECHOING=ECHO

ECHOS=ECHO

EDGE=SIDE

EDITED=EDIT

EDITING=EDIT

EDS=ED

EJECTING=EJECTED

ELIOTS=ELIOT

EMERGED=APPEAR

EMPHASISED=EMPHASISE

EMPHASISES=EMPHASISE

EMPHASISING=EMPHASISE

EMPLOYED=EMPLOY

EMU=ANIMAL

ENDED=END

ENDING=END

ENDURED=ENDURE

ENDURING=ENDURE

ENGINEERING=ENGINEERING

ENGLISHMEN=ENGLISHMAN

ENJOYED=ENJOY

ENQUIRED=ENQUIRE

ENQUIRING=ENQUIRE

ENSURED=ENSURE

ENTERED=ENTER

ENTERING=ENTER

ENTERTAINED=ENTERTAIN

ENTRANCE=ENTER

ENTRY=ENTER

EPILEPTIC=MEDICAL_CONDITION

EQUALLED=EQUAL

EQUALLING=EQUAL

EQUIPMENTS=EQUIPMENT

ERRATICALLY=ERRATIC

ESCAPED=ESCAPE

ESCAPING=ESCAPE

EUROPEANS=EUROPEAN

EVENING=EVENING

EVOKED=EVOKE

EVOKING=EVOKE

EXAMINED=EXAMINE

EXAMINING=EXAMINE

EXCEEDED=EXCEED

EXCHANGED=EXCHANGE

EXCHANGING=EXCHANGE

EXCLAIMED=EXCLAIM

EXCLAIMING=EXCLAIM

EXCLUDED=EXCLUDE

EXCLUDING=EXCLUDE

EXCUSED=EXCUSE

EXCUSES=EXCUSE

EXECRATED=ACCELERATOR

EXECUTED=EXECUTE

EXHIBITED=EXHIBIT

EXHIBITING=EXHIBIT

EXITED=EXIT

EXITING=EXIT

EXPANDED=EXPAND

EXPLAINED=EXPLAIN

EXPLODED=EXPLODE

EXPLODING=EXPLODE

EXPLOITED=EXPLOIT

EXPLOITING=EXPLOIT

EXPLORED=EXPLORE

EXPLORING=EXPLORE

EXPRESSING=EXPRESS

EXTRACTED=TRAPPED

EYES=EYE

FABRICS=FABRIC

FADED=FADE

FADING=FADE

FAILED=FAIL

FAILING=FAIL

FAINTER=FAINT

FAINTEST=FAINT

FAIRER=FAIR

FAIREST=FAIR

FALLEN=FALL

FALLING=FALL

FAMILY=FAMILY_OTHER

FAMILYS=FAMILY_OTHER

FARTHER=FAR

FARTHEST=FAR

FAST=SPEED

FASTER=FAST

FASTEST=FAST

FATHER=PARENT

FATHERS=PARENT

FATTER=FAT

FATTEST=FAT

FAULTY=MECHANICAL_FAILURE

FAVOURED=FAVOUR

FAVOURING=FAVOUR

FAVOURITES=FAVOURITE

FAVOURS=FAVOUR

FEATURED=FEATURE

FED=FEED

FEEDBACKS=FEEDBACK

FEELING=FEELING

FEET=FOOT

FELL=FALL

FELT=FEEL

FENCES=FENCE

FENCING=FENCE

FIBRES=FIBRE

FIERCER=FIERCE

FIERCEST=FIERCE

FIGHT=INTENTIONAL

FIGURED=FIGURE

FIGURING=FIGURE

FILED=FILE

FILING=FILE

FILLED=FILL

FILLING=FILL

FINDING=FIND

FINED=FINE

FINER=FINE

FINEST=FINE

FINING=FINE

FIRED=FIRE

FIREFIGHTERS=FIRST_RESPONDERS

FIREMAN=FIRST_RESPONDERS

FIREMAN=FIRST_RESPONDERS

FIRES=FIRE

FIRING=FIRE

FIRMER=FIRM

FIRMEST=FIRM

FISHERMEN=FISHERMAN

FISHING=FISHING

FISHTAILED=IMPACT_MOTION

FISHTAILING=IMPACT_MOTION

FITTER=FIT

FITTEST=FIT

FITTING=FIT

FLAMES=FIRE

FLATTER=FLAT

FLAVOURS=FLAVOUR

FLED=FLEE

FLEW=FLY

FLINGING=FLING

FLIPPED=ROLL

FLIPPING=FLIP

FLIPPING=ROLL

FLOATED=FLOAT

FLOATING=FLOAT

FLOODED=FLOOD

FLOODING=FLOOD

FLOWN=FLY

FLUNG=FLING

FLYING=FLY

FOCI=FOCUS

FOCUSED=FOCUS

FOCUSES=FOCUS

FOCUSING=FOCUS

FOCUSSED=FOCUS

FOCUSSES=FOCUS

FOCUSSING=FOCUS

FOG=VISIBILITY

FOGGY=VISIBILITY

FOLIAGE=TREE

FONDER=FOND

FONDEST=FOND

FONT=FRONT

FORA=FORUM

FORBADE=FORBID

FORBIDDEN=FORBID

FORCED=FORCE

FORCING=FORCE

FORD=MOTOR_VEHICLE

FORGAVE=FORGIVE

FORGIVEN=FORGIVE

FORGOT=FORGET

FORGOTTEN=FORGET

FOUGHT=FIGHT

FOUND=FIND

FOUNDED=FOUND

FOX=ANIMAL

FRAMED=FRAME

FRAMING=FRAME

FREED=FREE

FREER=FREE

FREEST=FREE

FREEWAY=HIGHWAY

FRESHER=FRESH

FRESHEST=FRESH

FRIDAYS=FRIDAY

FRIDGES=FRIDGE

FRO=FROM

FRON=FRONT

FROZE=FREEZE

FROZEN=FREEZE

FULFILLED=FULFIL

FULFILLING=FULFIL

FULFILS=FULFIL

FULLER=FULL

FULLEST=FULL

FUNDED=FUND

FURTHER=FAR

FURTHEST=FAR

FWY=HIGHWAY

GAINED=GAIN

GARAGE=CARPORT

GASES=GAS

GASP=GASP

GASPED=GASP

GASPING=GASP

GASSES=GAS

GATE=FENCE

GAVE=GIVE

GEARED=GEAR

GENIUSES=GENIUS

GENS=GEN

GENTLEMEN=GENTLEMAN

GERMANIES=GERMANY

GERMANS=GERMAN

GERMANYS=GERMANY

GETTIN=GET

GIBSONS=GIBSON

GIRLFRIEND=PARTNER

GIRLFRIENDS=GIRLFRIEND

GIVEAWAY=GIVEWAY

GIVEN=GIVE

GIVETH=GIVE

GLANCED=LOOK

GLARED=GLARE

GLARING=GLARE

GLOUCESTERS=GLOUCESTER

GOIN=GO

GOING=GO

GOLDS=GOLD

GONE=GO

GORDONS=GORDON

GOT=GET

GOTTEN=GET

GOVERNED=GOVERN

GRADER=ROADWORK

GRADIENT=EMBANKMENT

GRADING=ROADWORK

GRANDER=GRAND

GRANDEST=GRAND

GRANDKIDS=FAMILY_OTHER

GRASPED=GRASP

GRASPING=GRASP

GRASSED=GRASS

GRASSY=GRASS

GRATED=ROADWORK

GRAVEL=GRAVEL

GREASY=ROAD_CONDITIONS

GREATER=GREAT

GREATEST=GREAT

GREED=GREEN

GREEKS=GREEK

GREENER=GREEN

GREENEST=GREEN

GREENHOUSES=GREENHOUSE

GREETED=GREET

GREETING=GREET

GREW=GROW

GREYER=GREY

GRIEFS=GRIEF

GRIMMER=GRIM

GRIMMEST=GRIM

GRIP=HOLD

GRIPPED=HOLD

GROSSER=GROSS

GROWN=GROW

GUARANTEED=GUARANTEE

GUESSING=GUESS

GUIDED=GUIDE

GUILTS=GUILT

GUST=POOR_WEATHER

GUSTS=POOR_WEATHER

GUTTER=CURB

HAD=HAVE

HAIL=POOR_WEATHER

HAILING=POOR_WEATHER

HAIRPIN=BEND

HALFS=HALF

HALT=STATIONARY

HALTING=STATIONARY

HALVES=HALF

HAMILTONS=HAMILTON

HANDBRAKES=HANDBRAKE

HANDED=HAND

HANDLE=HANDLEBAR

HANDLEBAR=HANDLEBAR

HANDLEBARS=HANDLEBAR

HANDLING=HANDLING

HANDSOMEST=HANDSOME

HANGED=HANG

HANGING=HANG

HARASSED=INTENTIONAL

HARBOURS=HARBOUR

HARDER=HARD

HARDEST=HARD

HARRIES=HARRY

HARRISONS=HARRISON

HARSHER=HARSH

HARSHEST=HARSH

HARVEYS=HARVEY

HAS=HAVE

HATCHBACK=MOTOR_VEHICLE

HATREDS=HATRED

HAULED=HAUL

HAULING=HAUL

HAZARDS=WARN

HEADACHES=HEADACHE

HEADED=HEAD

HEADING=HEAD

HEADLIGHTS=HEADLIGHT

HEADY=HEAVY

HEARD=HEAR

HEARING=HEARING

HEATED=HEAT

HEATING=HEAT

HELD=HOLD

HELENS=HELEN

HELMUT=HELMET

HELPED=HELP

HELPING=HELP

HER=SHE

HERBERTS=HERBERT

HEREFORDS=HEREFORD

HEROES=HERO

HEROIN=SUBSTANCES

HEROS=HERO

HID=HIDE

HIDDEN=HIDE

HIGHER=HIGH

HIGHEST=HIGH

HIGHLIGHTED=HIGHLIGHT

HIGHWAY=HIGHWAY

HILL=HILL

HIM=HE

HIRED=HIRE

HIRING=HIRE

HIT=HIT

HITTING=HIT

HOLDEN=MOTOR_VEHICLE

HOLDING=HOLD

HOLDING=HOLD

HOLIEST=HOLY

HOLLANDS=HOLLAND

HONDA=MOTOR_VEHICLE

HONOURED=HONOUR

HONOURING=HONOUR

HONOURS=HONOUR

HOONING=INTENTIONAL

HOPED=HOPE

HOPING=HOPE

HOPPED=JUMP

HORN=WARN

HORSE=ANIMAL

HOTTER=HOT

HOTTEST=HOT

HOUSED=HOUSE

HOUSES=HOUSE

HOUSEWIVES=HOUSEWIFE

HOWARDS=HOWARD

HSB=PARTNER

HUMOURS=HUMOUR

HUMP=BUMP

HUNG=HANG

HUSBAND=PARTNER

HUSBANDS=PARTNER

HWY=HIGHWAY

HYDROPLANE=IMPACT_MOTION

HYPOTHESES=HYPOTHESIS

HYUNDAI=MOTOR_VEHICLE

ICE=POOR_WEATHER

ICY=POOR_WEATHER

IGNORED=IGNORE

IGNORING=IGNORE

ILLEGAL=INTENTIONAL

IMAGINED=IMAGINE

IMAGINING=IMAGINE

IMPACT=IMPACT

IMPACTED=IMPACT

IMPRESSING=IMPRESS

IMPULSES=IMPULSE

INCIDENCES=INCIDENCE

INCLINE=EMBANKMENT

INCLUDED=INCLUDE

INCLUDING=INCLUDE

INCOMING=ONCOMING

INCREASED=INCREASE

INCREASES=INCREASE

INCREASING=INCREASE

INCREASINGLY=INCREASE

INCURRING=INCUR

INDIANS=INDIAN

INDICATED=INDICATE

INDICATES=INDICATE

INDICATING=INDICATE

INDICATING=INDICATE

INDICATION=INDICATE

INDICATOR=INDICATE

INDICATOR=INDICATE

INDICES=INDEX

INDULGED=INDULGE

INDULGING=INDULGE

INFORMATIONS=INFORMATION

INFORNT=INFRONT

INHERITED=INHERIT

INHERITING=INHERIT

INHIBITED=INHIBIT

INHIBITING=INHIBIT

INJURE=INJURY

INJURED=INJURE

INJURING=INJURE

INJURY=INJURY

INSPIRED=INSPIRE

INSPIRING=INSPIRE

INSTALLED=INSTALL

INSTALLING=INSTALL

INSURANCES=INSURANCE

INSURED=INSURE

INTERFERED=INTERFERE

INTERFERING=INTERFERE

INTERPRETED=INTERPRET

INTERPRETING=INTERPRET

INTERRUPTED=INTERRUPT

INTERSECTIONS=INTERSECTION

INTERVENED=INTERVENE

INTERVENING=INTERVENE

INTOXICATED=SUBSTANCES

INVADED=INVADE

INVADING=INVADE

INVITED=INVITE

INVITING=INVITE

INVOKED=INVOKE

INVOKING=INVOKE

IS=BE

ITALIANS=ITALIAN

JACKSONS=JACKSON

JANES=JANE

JEAN=JEANS

JEANS=JEANS

JEWS=JEW

JOES=JOE

JOHNNIES=JOHNNY

JOHNSONS=JOHNSON

JOINED=JOIN

JOINING=JOIN

JOKED=JOKE

JOKING=JOKE

JORDANS=JORDAN

JOYCES=JOYCE

JUDGEMENTS=JUDGEMENT

JUMPED=JUMP

JUMPING=JUMP

JUMPS=JUMP

KARENS=KAREN

KATES=KATE

KEENER=KEEN

KEENEST=KEEN

KEEPING=KEEP

KENNEDYS=KENNEDY

KEPT=KEEP

KERB=CURB

KILLED=KILL

KILLING=KILL

KILOMETRES=KILOMETRE

KINDER=KIND

KINDEST=KIND

KINNOCKS=KINNOCK

KISSING=KISS

KMPH=SPEED

KMS=KM

KNELT=KNEEL

KNEW=KNOW

KNIVES=KNIFE

KNOCKED=KNOCK

KNOCKING=KNOCK

KNOWLEDGES=KNOWLEDGE

KNOWN=KNOW

KOREAS=KOREA

KPH=SPEED

LABELLED=LABEL

LABELLING=LABEL

LABOURS=LABOUR

LADEN=LOAD

LAID=LAY

LANCASTERS=LANCASTER

LANDED=LAND

LANDING=LAND

LANES=LANE

LARGER=LARGE

LARGEST=LARGE

LATER=LATE

LATEST=LATE

LAUGHED=LAUGH

LBS=LB

LEANED=LEAN

LEANING=LEAN

LEANT=LEAN

LEAPED=LEAP

LEAPING=LEAP

LEAPT=LEAP

LEARNED=LEARN

LEARNT=LEARN

LEASES=LEASE

LEAVES=LEAF

LED=LEAD

LEFT=LEAVE

LEFTS=LEFT

LENT=LEND

LEONARDS=LEONARD

LEVELLED=LEVEL

LICENCES=LICENCE

LIES=LIE

LIFES=LIFE

LIFESPANS=LIFESPAN

LIGHTED=LIGHT

LIGHTER=LIGHT

LIGHTEST=LIGHT

LIGHTPOLE=POLE

LIGHTS=LIGHT

LIKED=LIKE

LIKING=LIKE

LIMITED=LIMIT

LIMITING=LIMIT

LINED=LINE

LISTING=LISTING

LIT=LIGHT

LIVES=LIFE

LIVING=LIVE

LOADED=LOAD

LOADING=LOAD

LOCOMOTIVE=MOTOR_VEHICLE

LOGICS=LOGIC

LONDONS=LONDON

LONGED=LONG

LONGER=LONG

LONGEST=LONG

LONGING=LONG

LOOKED=LOOK

LOOKING=LOOK

LOSING=LOSE

LOSS=LOSE

LOST=LOSE

LOTHIANS=LOTHIAN

LOUDER=LOUD

LOUDEST=LOUD

LOWER=LOW

LOWEST=LOW

LSOE=LOSE

LUKES=LUKE

MACDONALDS=MACDONALD

MADDER=MAD

MADE=MAKE

MAINTAINED=MAINTAIN

MAKETH=MAKE

MAKING=MAKE

MALCOLMS=MALCOLM

MANUFACTURED=MANUFACTURE

MARIES=MARY

MARIJUANA=SUBSTANCES

MARKETED=MARKET

MARSHALLS=MARSHALL

MARYS=MARY

MAS=AMBULANCE

MASSES=MASS

MATRICES=MATRIX

MATURER=MATURE

MAXIMA=MAXIMUM

MAXWELLS=MAXWELL

MAYS=MAY

MAZDA=MOTOR_VEHICLE

MB=MOTORCYCLE

MBA=MOTORCYCLE

ME=I

MEANER=MEAN

MEANEST=MEAN

MEANT=MEAN

MEASURED=MEASURE

MECHANICS=MECHANIC

MEDIA=MEDIUM

MEMORANDA=MEMORANDUM

MEN=MAN

MENUS=MENU

MEREST=MERE

MERGED=MERGE

MERGING=MERGE

MET=MEET

METRES=METRE

MFB=FIRST_RESPONDERS

MICE=MOUSE

MICHAELS=MICHAEL

MICKS=MICK

MIDDLE=CENTRE

MILDER=MILD

MILDEST=MILD

MINDED=MIND

MINE=MY

MINIBUS=VAN

MINIMA=MINIMUM

MINIVAN=VAN

MISS=MISS

MISSED=MISS

MISSING=MISS

MISTAKE=ERROR

MISTAKEN=MISTAKE

MISTAKING=MISTAKE

MISTOOK=MISTAKE

MITCHELLS=MITCHELL

MLS=ML

MODELLED=MODEL

MODELLING=MODEL

MONARO=MOTOR_VEHICLE

MONDAYS=MONDAY

MONITORED=MONITOR

MONKEY_BIKE=MOTORCYCLE

MORALES=MORALE

MORNING=MORNING

MOSAICS=MOSAIC

MOTHER=PARENT

MOTORBIKE=MOTORCYCLE

MOTORBIKES=MOTORCYCLE

MOTORCROSS=MOTORCYCLE

MOTORHOME=VAN

MOTORIST=MOTOR_VEHICLE

MOTORWAYS=MOTORWAY

MOUND=BUMP

MOUNTED=MOUNT

MOUNTING=MOUNT

MOUSES=MOUSE

MOVED=MOVE

MOVIES=MOVIE

MOVING=MOVE

MOZARTS=MOZART

MPS=MP

MUCOSAE=MUCOSA

MUD=SOIL

MUDDY=SOIL

MUDS=MUD

MUM=PARENT

MURMURED=MURMUR

MURMURING=MURMUR

MURRAYS=MURRAY

MUSICS=MUSIC

MV=MOTOR_VEHICLE

MVA=ACCIDENT

NAMED=NAME

NAMING=NAME

NARROWER=NARROW

NARROWEST=NARROW

NEARER=NEAR

NEAREST=NEAR

NEATER=NEAT

NEATEST=NEAT

NEEDED=NEED

NEIGHBOURHOODS=NEIGHBOURHOOD

NEIGHBOURS=NEIGHBOUR

NEWER=NEW

NEWEST=NEW

NEWTONS=NEWTON

NICER=NICE

NICEST=NICE

NIL=NONE

NORMANS=NORMAN

NOS=NO

NOSE=IMPACT_MOTION

NOSEDIVE=IMPACT_MOTION

NOTED=NOTE

NOTING=NOTE

NURSERY=NURSERY

OBEYED=OBEY

OBSCURED=OBSCURE

OBSCURING=OBSCURE

OBSERVE=SEE

OBSERVED=SEE

OBTAINED=OBTAIN

OCCUPANTS=OCCUPANT

OCCURRED=HAPPENED

OCCURRING=OCCUR

ODYSSEY=MOTOR_VEHICLE

OFFENCES=OFFENCE

OFFEND=FAULT

OFFENDING=FAULT

OFFERING=OFFER

OIL=SLIP

OILY=SLIP

OKAY=OK

OLDER=OLD

OLDEST=OLD

ON_TOP=ONTOP

OPENED=OPEN

OPPOSING=OPPOSITE

OPTED=OPT

ORANGE=AMBERLIGHT

ORGANISATIONS=ORGANISATION

ORGANISED=ORGANISE

ORGANISERS=ORGANISER

ORGANISES=ORGANISE

ORGANISING=ORGANISE

OUTLINED=OUTLINE

OVERCAME=OVERCOME

OVERCOMING=OVERCOME

OVERCORRECTED=OVERCORRECT

OVERLOOKED=OVERLOOK

OVERLOOKING=OVERLOOK

OVERSHOT=OVERCORRECT

OVERSHOT=OVERCORRECT

OVERTAKING=OVERTAKE

OVERTOOK=OVERTAKE

OVERTURNED=ROLL

OWED=OWE

OWES=OWE

OWING=OWE

OXFORDS=OXFORD

OXYGENS=OXYGEN

PAID=PAY

PALER=PALE

PALEST=PALE

PANICS=PANIC

PARADE=ROADWAY

PARAS=PARA

PARENTS=PARENT

PARKED=PARK

PARKING=PARK

PARTED=PART

PARTNER=PARTNER

PAS=PASSENGER

PASSANGER=PASSENGER

PASSED=OVERTAKE

PASSENGERS=PASSENGER

PASSING=PASS

PATIENTS=PATIENT

PAULS=PAUL

PAUSE=STATIONARY

PAUSED=PAUSE

PAUSED=STATIONARY

PAUSES=PAUSE

PAVEMENT=FOOTPATH

PCS=PC

PDE=ROADWAY

PED=PEDESTRIAN

PEDESTRIANS=PEDESTRIAN

PERFORMED=PERFORM

PERFORMING=PERFORM

PERSUADED=PERSUADE

PERSUADING=PERSUADE

PHASES=PHASE

PHENOMENA=PHENOMENON

PHONED=PHONE

PHONING=PHONE

PHRASES=PHRASE

PICTURED=PICTURE

PIES=PIE

PILED=PILE

PILING=PILE

PILLAR=POLE

PILLION=MOTORCYCLIST

PINKER=PINK

PINNED=TRAPPED

PINNING=TRAPPED

PITT=EMBANKMENT

PL=ROADWAY

PLACE=ROADWAY

PLAINER=PLAIN

PLAINEST=PLAIN

PLASTICS=PLASTIC

PLCS=PLC

PLEADED=PLEAD

PLEASANTER=PLEASANT

PLEASANTEST=PLEASANT

PLEASES=PLEASE

PLOUGHED=CRASH

PLOWED=CRASH

PLUNGED=PLUNGE

PLUNGING=PLUNGE

POLARIS=MOTOR_VEHICLE

POLES=POLE

POLICEMAN=POLICE

POLICEMEN=POLICE

POLITEST=POLITE

POLL=POLE

POLYTECHNICS=POLYTECHNIC

POORER=POOR

POOREST=POOR

POSSESSING=POSSESS

POSTPONED=POSTPONE

POTATOES=POTATO

POTHOLE=ROAD_CONDITIONS

POURED=POUR

POWERPOLE=POLE

POWERPOLE=POLE

PRECEDED=PRECEDE

PRECEDING=PRECEDE

PREFERRING=PREFER

PREPARED=PREPARE

PREPARING=PREPARE

PRESSING=PRESS

PRIEST=PRIEST

PRIVATISATIONS=PRIVATISATION

PROCEEDED=PROCEED

PROCEEDING=APPROACH

PROCESSING=PROCESS

PROCLAIMED=PROCLAIM

PROCLAIMING=PROCLAIM

PROFOUNDER=PROFOUND

PROFOUNDEST=PROFOUND

PROGRAMMES=PROGRAMME

PROGRESSING=PROGRESS

PROHIBITED=PROHIBIT

PROHIBITING=PROHIBIT

PROMOTED=PROMOTE

PROMOTING=PROMOTE

PROMPTED=PROMPT

PROPELLING=AIRBORNE

PROSECUTED=PROSECUTE

PROUDER=PROUD

PROUDEST=PROUD

PROVEN=PROVE

PROVIDED=PROVIDE

PROVOKED=PROVOKE

PROVOKING=PROVOKE

PS=P

PT=PATIENT

PTS=PATIENT

PUBLICS=PUBLIC

PULLED=PULL

PULLING=PULL

PULSE=PULSE

PULSES=PULSE

PURCHASES=PURCHASE

PURER=PURE

PUREST=PURE

PURSUING=PURSUE

PURSUIT=PURSUE

PUSHING=PUSH

PUTTED=PUT

PYLON=POLE

PYLORI=PYLORUS

QUAD=MOTORCYCLE

QUADBIKE=MOTORCYCLE

QUICKER=QUICK

QUIETER=QUIET

QUIETEST=QUIET

QUITTED=QUIT

QUOTED=QUOTE

QUOTING=QUOTE

RABBIT=ANIMAL

RAILING=BARRIER

RAILWAY=TRAIN

RAIN=POOR_WEATHER

RAINED=RAIN

RAINING=POOR_WEATHER

RAINY=POOR_WEATHER

RALPHS=RALPH

RAMPS=RAMP

RAN=RUN

RANG=RING

RANGED=RANGE

RANGER=MOTOR_VEHICLE

RANGING=RANGE

RARER=RARE

RAREST=RARE

RATING=RATING

RAVINE=EMBANKMENT

RD=ROAD

READING=READ

REALISED=REALISE

REALISES=REALISE

REALISING=REALISE

REAREND=IMPACT_MOTION

REASSURED=REASSURE

REBUILT=REBUILD

RECALLED=RECALL

RECALLING=RECALL

RECKONED=RECKON

RECKONING=RECKON

RECOGNISED=RECOGNISE

RECOGNISES=RECOGNISE

RECOGNISING=RECOGNISE

RECRUITED=RECRUIT

RECRUITING=RECRUIT

REDDER=RED

REFERENDA=REFERENDUM

REFERRING=REFER

REFS=REF

REFUSED=REFUSE

REFUSES=REFUSE

REGAINED=REGAIN

RELATIVE=FAMILY_OTHER

RELATIVES=FAMILY_OTHER

RELEASES=RELEASE

RELIEFS=RELIEF

REMAINED=REMAIN

REMINDED=REMIND

REMOTER=REMOTE

REMOTEST=REMOTE

REPAID=REPAY

REPAIRED=REPAIR

REPAIRING=REPAIR

REPEATED=REPEAT

REPEATING=REPEAT

REPUBLICS=REPUBLIC

REQUIRED=REQUIRE

REQUIRING=REQUIRE

RESPONDED=RESPOND

RESTORING=RESTORE

RESULTED=RESULT

RESULTING=RESULT

RETAINED=RETAIN

RETIRED=RETIRE

RETIRING=RETIRE

RETURNED=RETURN

REVERSED=REVERSE

REVERSING=REVERSE

REVS=REV

REVVED=REV

RICHARDSONS=RICHARDSON

RICHER=RICH

RICHEST=RICH

RICOCHETED=THROW

RIDDEN=RIDE

RIDER=RIDER

RIDERS=RIDER

RINGING=RING

RISE=RIDGE

RISEN=RISE

RISKING=RISK

ROADBIKE=PEDAL_BIKE

ROADS=ROAD

ROADSIDE=ROAD

ROADWORKS=ROADWORK

ROARED=ROAR

ROARING=ROAR

ROCKS=ROCK

ROCKY=ROCK

RODE=RIDE

ROLLED=ROLL

ROLLING=ROLL

ROLLING=ROLL

ROLLOVER=ROLL

ROMANS=ROMAN

ROOM=SPACE

ROOT=TREE

ROOTED=ROOT

ROOTS=TREE

ROSE=RISE

ROUGHER=ROUGH

ROUGHEST=ROUGH

ROUNDED=ROUND

ROUNDER=ROUND

RUDER=RUDE

RUDEST=RUDE

RULED=RULE

RUMOURS=RUMOUR

RUNG=RING

RUNNIN=RUN

RUNNINGS=RUNNING

RUSSELLS=RUSSELL

RUSSIANS=RUSSIAN

RUT=ROAD_CONDITIONS

SADDER=SAD

SADDEST=SAD

SAFER=SAFE

SAFEST=SAFE

SAID=SAY

SAND=SOIL

SANG=SING

SANK=SINK

SAT=SIT

SATURDAYS=SATURDAY

SAVING=SAVE

SAW=SEE

SAYED=SAY

SAYIN=SAY

SCHEDULED=SCHEDULE

SCHEDULING=SCHEDULE

SCORED=SCORE

SCORING=SCORE

SCOTS=SCOT

SCOTTS=SCOTT

SCRUB=TREE

SEATED=SEAT

SEATING=SEAT

SECURED=SECURE

SECURING=SECURE

SEDAN=MOTOR_VEHICLE

SEEKING=SEEK

SEEMED=SEEM

SEEMING=SEEM

SEEN=SEE

SEIZURE=MEDICAL_CONDITION

SELLING=SELL

SELVES=SELF

SEMI-TRAILER=HEAVY_VEHICLE

SENT=SEND

SERVED=VEER

SES=FIRST_RESPONDERS

SETA=SEAT

SETTING=SET

SEVEREST=SEVERE

SHAKEN=SHAKE

SHAKING=SHAKE

SHALLOWER=SHALLOW

SHALLOWEST=SHALLOW

SHAPED=SHAPE

SHAPING=SHAPE

SHARED=SHARE

SHARING=SHARE

SHEEP=ANIMAL

SHELVES=SHELF

SHINED=SHINE

SHINING=SHINE

SHITS=SHIT

SHOES=SHOE

SHONE=SHINE

SHOOK=SHAKE

SHOPPING=SHOPPING

SHORTER=SHORT

SHORTEST=SHORT

SHOT=SHOOT

SHOUTED=SHOUT

SHOUTING=SHOUT

SHOWN=SHOW

SHYER=SHY

SICKER=SICK

SIDESWIPE=SWIPE

SIGHED=SIGH

SIGNALLED=INDICATE

SIGNALLED=SIGNAL

SIGNALLING=SIGNAL

SIMPLER=SIMPLE

SIMPLEST=SIMPLE

SINGIN=SING

SINGING=SING

SISTER=SIBLING

SISTERS=SIBLING

SITTING=SIT

SKIDDED=SKID

SKIDDING=SKID

SLEEP=FATIGUE

SLEEPING=SLEEP

SLEPT=SLEEP

SLID=SLIDE

SLID=SLIDE

SLIDING=SLIDE

SLIGHTER=SLIGHT

SLIGHTEST=SLIGHT

SLIMMER=SLIM

SLIMMEST=SLIM

SLIPPED=SLIP

SLIPPERY=SLIP

SLOWDOWN=SLOW

SLOWED=SLOW

SLOWER=SLOW

SLOWEST=SLOW

SLOWING=SLOW

SLOWLY=SLOW

SMA=SAME

SMALLER=SMALL

SMALLEST=SMALL

SMARTER=SMART

SMARTEST=SMART

SMASHED=CRASH

SMELLED=SMELL

SMELLING=SMELL

SMELT=SMELL

SMILED=SMILE

SMILING=SMILE

SMOKE=MECHANICAL_FAILURE

SMOKED=SMOKE

SMOKING=SMOKE

SMOOTHED=SMOOTH

SMOOTHER=SMOOTH

SMOOTHEST=SMOOTH

SOFTER=SOFT

SOFTEST=SOFT

SOIL=SOIL

SOLD=SELL

SOMERSAULT=AIRBORNE

SON=OFFSPRING

SOOT=SLIP

SOUGHT=SEEK

SOUNDED=SOUND

SOUNDER=SOUND

SOUNDEST=SOUND

SPARED=SPARE

SPARING=SPARE

SPAT=SPIT

SPEAKING=SPEAK

SPECIALISED=SPECIALISE

SPECIALISES=SPECIALISE

SPECIALISING=SPECIALISE

SPECTRA=SPECTRUM

SPED=SPEED

SPEEDED=SPEED

SPEEDING=SPEED

SPEEDO=SPEED

SPELLED=SPELL

SPELT=SPELL

SPENCERS=SPENCER

SPENDING=SPENDING

SPENT=SPEND

SPILLED=SPILL

SPILLING=SPILL

SPILT=SPILL

SPINNING=SPIN

SPOILT=SPOIL

SPOKE=SPEAK

SPOKEN=SPEAK

SPOKESMEN=SPOKESMAN

SPONSORED=SPONSOR

SPONSORING=SPONSOR

SPRANG=SPRING

SPRINGING=SPRING

SPRUNG=SPRING

SPUN=SPIN

SQUASHED=TRAPPED

ST=STREET

STADIA=STADIUM

STAIR=STAIRS

STAIRCASES=STAIRCASE

STAIRS=STAIRS

STALLED=MECHANICAL_FAILURE

STALLING=MECHANICAL_FAILURE

STANDING=STAND

STANDSTILL=STATIONARY

STANLEYS=STANLEY

STARED=STARE

STARING=STARE

STARTED=START

STATIONERY=STATIONARY

STAVES=STAFF

STEAL=STOLEN

STEEPER=STEEP

STEEPEST=STEEP

STEERED=STEER

STEERING=STEER

STEP=WALK

STEPPED=WALK

STEWARTS=STEWART

STIFFER=STIFF

STIFFEST=STIFF

STIMULI=STIMULUS

STIRLINGS=STIRLING

STIRRING=STIR

STOLE=STEAL

STOLEN=STEAL

STONE=ROCK

STONES=GRAVEL

STOOD=STAND

STOPPED=STATIONARY

STORED=STORE

STORING=STORE

STORM=POOR_WEATHER

STRAIGHTEN=STRAIGHT

STRAIGHTENING=STRAIGHT

STRAIGHTER=STRAIGHT

STRAIGHTEST=STRAIGHT

STRAINED=STRAIN

STRANGEST=STRANGE

STREET=STREET

STRESSING=STRESS

STRICTER=STRICT

STRICTEST=STRICT

STRIKE=STRIKE

STRIKING=STRIKE

STRIVEN=STRIVE

STRODE=STRIDE

STROKED=STROKE

STROKING=STROKE

STRONGER=STRONG

STRONGEST=STRONG

STROVE=STRIVE

STRUCK=STRIKE

STUARTS=STUART

STUCK=STICK

STUCK=TRAPPED

STUMP=ROCK

SUBSTITUTED=SUBSTITUTE

SUCCEEDED=SUCCEED

SUDDEN=SUDDENLY

SUEING=SUE

SUFFERING=SUFFER

SUITED=SUIT

SUITING=SUIT

SUMMARISED=SUMMARISE

SUMMARISES=SUMMARISE

SUMMARISING=SUMMARISE

SUMMONED=SUMMON

SUMMONING=SUMMON

SUNDAYS=SUNDAY

SUNG=SING

SUNGLARE=SUN_GLARE

SUNK=SINK

SUPPLIER=SUPPLIER

SUPPRESSING=SUPPRESS

SURER=SURE

SUREST=SURE

SURFACE=ROAD_SURFACE

SURROUNDED=SURROUND

SURVEYED=SURVEY

SUSTAINED=SUSTAIN

SUV=MOTOR_VEHICLE

SWAM=SWIM

SWEEPING=SWEEP

SWEETER=SWEET

SWEETEST=SWEET

SWELLED=SWELL

SWELLING=SWELL

SWEPT=SWEEP

SWERVE=VEER

SWERVED=VEER

SWERVING=VEER

SWINGING=SWING

SWIPED=SWIPE

SWOLLEN=SWELL

SWORE=SWEAR

SWORN=SWEAR

SWUM=SWIM

SWUNG=SWING

SYNTHESES=SYNTHESIS

TACTICS=TACTIC

TAKEN=TAKE

TAKING=TAKE

TALKIN=TALK

TALLER=TALL

TALLEST=TALL

TANKER=HEAVY_VEHICLE

TAP=CLIPPED

TARAGO=MOTOR_VEHICLE

TARGETED=TARGET

TARGETING=TARGET

TARGETTED=TARGET

TARGETTING=TARGET

TASK=TASK

TASTED=TASTE

TASTING=TASTE

TAUGHT=TEACH

TAXIS=TAXI

TAYLORS=TAYLOR

TBONED=TBONE

TBONES=TBONE

TEETH=TOOTH

TELEPHONED=TELEPHONE

TELEPHONING=TELEPHONE

TELLIN=TELL

TELLING=TELL

TEMPTED=TEMPT

TESTED=TEST

THEATRES=THEATRE

THEE=THOU

THEIRSELVES=THEMSELVES

THEM=THEY

THESES=THESIS

THICKER=THICK

THIEVES=THIEF

THING=THING

THINNER=THIN

THINNEST=THIN

THOMPSONS=THOMPSON

THOUGHT=THINK

THREW=THROW

THROWING=THROW

THROWN=THROW

THROWN=THROW

THRU=THROUGH

THURSDAYS=THURSDAY

TIED=TIE

TIES=TIE

TIGHTER=TIGHT

TIGHTEST=TIGHT

TIMED=TIME

TIMES=TIME

TIPPED=ROLL

TIRE=TYRE

TIRED=FATIGUE

TOES=TOE

TOLD=TELL

TOMATOES=TOMATO

TOMMIES=TOMMY

TOMS=TOM

TONNES=TONNE

TOOK=TAKE

TOPICS=TOPIC

TOPPLE=FALL

TORE=TEAR

TORIES=TORY

TORN=TEAR

TOSS=TOSS

TOSSED=THROW

TOSSING=TOSS

TOTALLED=TOTAL

TOTALLING=TOTAL

TOUGHER=TOUGH

TOUGHEST=TOUGH

TOURED=TOUR

TOW=TOWING

TOWARDS=TOWARD

TOWBALL=TOWBALL

TOWED=TOWING

TOWED=TOWING

TOWING=TOWING

TOYOTA=MOTOR_VEHICLE

TRACKS=RAIL_TRACK

TRACTION=HOLD

TRADED=TRADE

TRAILERS=TOWING

TRAILOR=TOWING

TRAILS=TRACK

TRAINED=TRAIN

TRAINING=TRAINING

TRAINLINE=TRAIN

TRAMS=TRAM

TRANSFERRING=TRANSFER

TRAVELING=TRAVEL

TRAVELLED=TRAVEL

TRAVELLERS=TRAVELLER

TRAVELLING=TRAVEL

TREATED=TREAT

TREATING=TREAT

TREES=TREE

TRESS=TREE

TROUSER=TROUSERS

TROUSERS=TROUSERS

TRUCK=HEAVY_VEHICLE

TRUCKS=HEAVY_VEHICLE

TRUCKS=HEAVY_VEHICLE

TRUER=TRUE

TRUEST=TRUE

TUESDAYS=TUESDAY

TUMBLED=FALL

TUMBLING=FALL

TUMOURS=TUMOUR

TUNING=TURNING

TUNNEL=BRIDGE

TURNED=TURN

TURNS=TURN

TYING=TIE

TYPED=TYPE

TYPING=TYPE

TYRES=TYRE

TYRES=TYRE

UNCLE=FAMILY_OTHER

UNCLES=FAMILY_OTHER

UNDERGOING=UNDERGO

UNDERGONE=UNDERGO

UNDERLINED=UNDERLINE

UNDERMINED=UNDERMINE

UNDERMINING=UNDERMINE

UNDERSTANDING=UNDERSTANDING

UNDERSTOOD=UNDERSTAND

UNDERTAKEN=UNDERTAKE

UNDERTOOK=UNDERTAKE

UNDERWENT=UNDERGO

UNITING=UNITE

UNIXES=UNIX

UNLIKELIEST=UNLIKELY

UNREGISTERED=INTENTIONAL

US=WE

USED=USE

USES=USE

UTE=MOTOR_VEHICLE

VAST=VAST

VASTER=VAST

VEERED=VEER

VEERING=VEER

VEH=MOTOR_VEHICLE

VEHICLE=MOTOR_VEHICLE

VEHICLES=MOTOR_VEHICLE

VEHICLES=VEHICLE

VENTURED=VENTURE

VERSE=VERSUS

VERSES=VERSUS

VIRUSES=VIRUS

VISITED=VISIT

VISITING=VISIT

VOLS=VOL

VOTED=VOTE

VOTING=VOTE

VS=VERSUS

WAGON=MOTOR_VEHICLE

WAITED=WAIT

WAITING=WAIT

WAKE=FATIGUE

WAKING=WAKE

WALKED=WALK

WALKING=WALK

WALLABY=KANGAROO

WAN=WANT

WANTIN=WANT

WAREHOUSES=WAREHOUSE

WARMER=WARM

WARMEST=WARM

WARNED=WARN

WARNING=WARN

WARNING=WARNING

WASH=WASH

WASHING=WASHING

WASHINGTONS=WASHINGTON

WASHOUT=POOR_WEATHER

WASTED=WASTE

WASTING=WASTE

WD=MOTOR_VEHICLE

WEAKER=WEAK

WEDDING=WEDDING

WEDGED=TRAPPED

WEDNESDAYS=WEDNESDAY

WEEPING=WEEP

WEIGHED=WEIGH

WELCOMED=WELCOME

WELCOMING=WELCOME

WENT=GO

WEPT=WEEP

WERE=BE

WETTER=WET

WETTEST=WET

WHEATS=WHEAT

WHEELS=WHEEL

WHITER=WHITE

WHITEST=WHITE

WIDE=OVERCORRECT

WIDER=WIDE

WIDEST=WIDE

WIFE=PARTNER

WIFES=PARTNER

WILDER=WILD

WILDEST=WILD

WILE=WHILE

WILSONS=WILSON

WIND=POOR_WEATHER

WINDED=WIND

WINDSHIELD=WINDSCREEN

WINDY=POOR_WEATHER

WIPED=WIPE

WIPING=WIPE

WISER=WISE

WITHDRAWN=WITHDRAW

WITHDREW=WITHDRAW

WITNESSED=WITNESS

WITNESSES=WITNESS

WITNESSING=WITNESS

WIVES=WIFE

WOBBLE=SHAKY

WOBBLING=SHAKY

WOKEN=FATIGUE

WOKEN=WOKE

WOLVES=WOLF

WOMBAT=ANIMAL

WOMEN=WOMAN

WON=WIN

WORE=WEAR

WORK=WORK

WORKER=WORK

WORKERS=WORK

WORKFORCES=WORKFORCE

WORKING=WORK

WORKING=WORK

WORN=WEAR

WORSE=BAD

WORST=BAD

WOUNDED=WOUND

WOVE=WEAVE

WOVEN=WEAVE

WRITING=WRITE

WRITTEN=WRITE

WROTE=WRITE

WROUGHT=WORK

YA=YOU

YANKED=PULL

YAWN=FATIGUE

YAWNING=FATIGUE

YE=YOU

YELLED=YELL

YELLING=YELL

YER=YOUR

YOUNGER=YOUNG

YOUNGEST=YOUNG
