# Supplementary material for: Classification of road traffic injury collision characteristics using text mining analysis: Implications for road injury prevention
Source: PLoS One. 2021 Jan 27;16(1):e0245636. doi: 10.1371/journal.pone.0245636 (PMC7840051; doi:10.1371/journal.pone.0245636)
Supplement: S3 File — (DOCX) [file pone.0245636.s003.docx]

S3 File: Categorisation dictionary

ABRUPTLY

ABRUPT (1)

AT_THE_LAST_MINUTE (1)

SUDDEN (1)

SUDDENLY (1)

ACCELERATED

ACCELERATED (1)

TAKE_OFF (1)

ACCELERATOR (1)

ACCIDENTALLY (1)

ACCIDENTALLY_ACCELERATED

ACCIDENTALLY_ACCELERATED (1)

ACCIDENTALLY_HIT_THE_ACCELERATOR (1)

ASSUME_I_HIT_THE_ACCELERATOR (1)

HIT_THE_ACCELERATOR_INSTEAD_OF_THE_BRAKE (1)

ACROSS (1)

ACROSS_ROAD (1)

AMBERLIGHT

AMBER (1)

ORANGE (1)

ANIMALS

ANIMAL (1)

BIRD (1)

CAT (1)

COW (1)

DOG (1)

ECHIDNA (1)

EMU (1)

FOX (1)

HORSE (1)

RABBIT (1)

SHEEP (1)

WOMBAT (1)

ANOTHER (1)

ANOTHER_PARTY

@ANOTHERPARTY1 [ANOTHER BEFORE MOTOR_VEHICLE /A /S3] (1)

@ANOTHERPARTY2 [OTHER BEFORE MOTOR_VEHICLE /A /S3] (1)

A_CAR (1)

A_MOTOR_VEHICLE (1)

A_TRUCK (1)

ANOTHER_CAR (1)

ANOTHER_MOTOR (1)

ANOTHER_MOTORCYCLE (1)

ANOTHER_MOTORCYCLIST (1)

ANOTHER_PARTY (1)

ANOTHER_RIDER (1)

ANOTHER_VEHICLE (1)

OTHER_PARTY (1)

APPROACH (1)

AROUND (1)

AROUND_BEND

AROUND_A_BEND (1)

AROUND_A_BEND_AND_HIT (1)

AROUND_BEND (1)

AROUND_THE_BEND (1)

ON_BEND (1)

AROUND_CORNER

AROUND_A_CORNER (1)

AROUND_CORNER (1)

AROUND_THE_CORNER (1)

HAIRPIN (1)

TURN_CORNER (1)

AVENUE (1)

AVOID (1)

BACK_WHEEL (1)

BALANCE (1)

BARRIER

BARRIER (1)

BOLLARD (1)

BRIDGE_RAIL (1)

CONCRETE_BANNER (1)

DIVIDER (1)

RAILING (1)

BEHIND (1)

BENCH (1)

BEND

BEND (1)

WINDING_ROAD (1)

BIKE

@BICYCLE [BICYCLE NOT AFTER MOTOR /A /S1] (1)

BIKE (1)

PUSH_BIKE (1)

BLACKOUT

BLACKED_OUT (1)

BLACKING_OUT (1)

BLACKOUT (1)

BLANK_OUT (1)

BLANKED_OUT (1)

LOSE_CONSCIOUSNESS (1)

LOST_CONSCIOUSNESS (1)

PASS_OUT (1)

PASSED_OUT (1)

BONNET (1)

BOOT (1)

BRAKE

BRAKE (1)

THE_BREAK (1)

THE_BREAKS (1)

BRAKE_SUDDENLY

@BRAKESUDDENLY1 [BRAKE BEFORE SUDDENLY /A /S1] (1)

@BRAKESUDDENLY2 [BRAKE AFTER SUDDENLY /A /S1] (1)

@BS1 [STOP NEAR ABRUPTLY /A /S3] (1)

BRAKE_ABRUPT (1)

BRAKE_HARD (1)

BRAKE_HEAVILY (1)

BRAKE_SUDDENLY (1)

BREAK_SUDDENLY (1)

HIT_THE_BRAKE (1)

SLAM_THE_BRAKE (1)

SUDDENLY_BRAKE (1)

BUMP

BUMP (1)

SPEEDBUMP (1)

BUS (1)

BY (1)

BY_A (1)

BY_ANOTHER (1)

CARPARK

@CARPARK [MOTOR_VEHICLE BEFORE PARK /Y /S1] (1)

@CARPARK2 [MOTOR_VEHICLE BEFORE SPOT /A /S1] (1)

@CARPARK3 [MOTOR_VEHICLE BEFORE SPACE /A /S1] (1)

@CARPARK4 [PARK BEFORE SPACE /A /S1] (1)

@CARPARK5 [PARK BEFORE SPOT /A /S1] (1)

CARPARK (1)

CAUSE (1)

CAUSE_ME (1)

CHANGE (1)

CHANGE_LANE

CHANGE_LANE (1)

CHANGE_THE_LANE (1)

CHOCK (1)

CLIENT

CLIENT (1)

PATIENT (1)

PT (1)

VICTIM (1)

COLLIDE_WITH

COLLIDE_INTO (1)

COLLIDE_WITH (1)

COLLIDED_WITH (1)

COLLISION_WITH (1)

COLLISION

ACCIDENT (1)

COLLISION (1)

CRASH (1)

COME (1)

CORNER (1)

CROSS (1)

CULVERT (1)

CUT (1)

CUT_OFF

@CUTOFF1 [CUT BEFORE OFF /A /S3] (1)

CUT_OFF (1)

CYCLIST (1)

DELIBERATE_ACT

ASSAULT (1)

DELIBERATE_ACT (1)

DIDNT

DID_NOT (1)

DIDNT (1)

DIRECTION (1)

DIRT_ROAD

DIRT_ROAD (1)

DIRT_TRACK (1)

GRAVEL_ROAD (1)

DOOR (1)

DRAG (1)

DRIVE (1)

DRIVER (1)

DRIVER_COLLIDE (1)

DRIVEWAY

DRIVE_WAY (1)

DRIVEWAY (1)

RESIDENCE (1)

DRIVING (1)

EARTH_MATTER

BEACH (1)

DEBRIS (1)

DIRT (1)

DUNE (1)

EARTH (1)

MUD (1)

SAND (1)

SOIL (1)

EMBANKMENT

DEPRESSION (1)

DITCH (1)

DRAIN (1)

EMBANKMENT (1)

GRADIENT (1)

INCLINE (1)

PITT (1)

RAVINE (1)

ERODED (1)

EXIT

EXIT (1)

GET_OUT (1)

FAIL (1)

FAIL_TO_GIVE_WAY

DID_NOT_GIVE_WAY (1)

DID_NOT_GIVEWAY (1)

DIDNT_GIVEWAY (1)

FAIL_TO_GIVE_WAY (1)

FAIL_TO_GIVEWAY (1)

FALL

COM_OFF (1)

COME_OFF (1)

FALL (1)

THROW (1)

TOPPLE (1)

TUMBLE (1)

FALL_AFTER_HIT

@FALLAFTER [FALL AFTER HIT /A /S20] (1)

@FALLAFTER2 [FALL AFTER HIT_BY /A /S20] (1)

@FALLAFTER3 [FALL AFTER HIT_ME /A /S20] (1)

@FALLAFTER4 [FALL AFTER HIT_MY /A /S20] (1)

@FALLAFTER5 [FALL AFTER REVERSE /A /S20] (1)

@FALLAFTER6 [FALL AFTER BACK_MOTOR_VEHICLE_OUT /A /S20] (1)

@FALLAFTER7 [FALL AFTER ROLL /A /S20] (1)

FALL_ASLEEP (1)

FATIGUE

DOZED_OFF (1)

DRIVER_FALL_ASLEEP (1)

FALL_ASLEEP_AT_THE_WHEEL (1)

FALL_ASLEEP_AT_WHEEL (1)

FALL_ASLEEP_BEHIND (1)

FALL_ASLEEP_DRIVING

FALL_ASLEEP_WHILE_DRIVING (1)

SLEEP (1)

TIRED (1)

WOKE (1)

YAWN (1)

FENCE

FENCE (1)

GATE (1)

FOOT (1)

FOOTPATH

@PATH2 [PATH NOT BEFORE OF /A /S2] (1)

FOOTPATH (1)

FREEWAY

FREEWAY (1)

HIGHWAY (1)

FROM (1)

FROM_BEHIND (1)

FROM_SIDE (1)

FRONT (1)

GIVE_WAY

GIVE_WAY (1)

GIVEWAY (1)

GIVEWAY_SIGN

GIVE_WAY_SIGN (1)

GIVEWAY_SIGN (1)

GO (1)

GO_OVER (1)

GO_THROUGH (1)

GOT_OUT (1)

GRAVEL

GRAVEL (1)

UNMADE_ROAD (1)

GREEN (1)

GREEN_ARROW (1)

GREEN_LIGHT (1)

GREEN_MAN (1)

HAIL (1)

HAND_BRAKE

HAND_BRAKE (1)

HAND_BREAK (1)

HANDBRAKE (1)

HANDLEBAR (1)

HEAD (1)

HEAD_ON

FRONTAL (1)

HEAD_ON (1)

HEAVY_VEHICLE

COMMERCIAL_TRAILER (1)

GARBAGE_TRUCK (1)

PRIME_MOVER (1)

SEMI_TRAILER (1)

TANKER (1)

TRUCK (1)

HILL (1)

HIS (1)

HIT

BRUSH (1)

CLIP (1)

COLLECTED (1)

COLLIDE (1)

CRUSH (1)

HIT (1)

KNOCK (1)

SLAM (1)

SMASH (1)

STRUCK (1)

TAP (1)

HIT_AND_RUN

@HITRUN1 [TAKE_OFF AFTER DRIVER /A /S1] (1)

@HITRUN2 [TAKE_OFF AFTER RIDER /A /S1] (1)

@HITRUN3 [RUN AFTER HIT /A /S3] (1)

DROVE_OFF (1)

FLED_FROM_SCENE (1)

HIT_AND_RAN (1)

HIT_BY

CLIP_BY (1)

COLLECTED_BY (1)

COLLIDE_BY (1)

CRUSH_BY (1)

HAVE_BE_HIT (1)

HIT_BY (1)

HIT_PATIENT (1)

KNOCK_BY (1)

SLAM_BY (1)

SMASH_BY (1)

STRUCK_BY (1)

STUCK_BY (1)

WAS_HIT (1)

HIT_CURB

@HITCURB1 [CURB NEAR HIT /A /S4] (1)

@HITCURB2 [CURB AFTER GO_INTO /A /S4] (1)

@HITCURB3 [CURB AFTER GO_UP /A /S4] (1)

@HITCURB4 [GUTTER NEAR HIT /A /S4] (1)

@HITCURB5 [GUTTER AFTER GO_INTO /A /S4] (1)

@HITCURB6 [GUTTER AFTER GO_UP /A /S4] (1)

@HITCURB7 [GUTTER AFTER OVER /A /S4] (1)

@HITCURB8 [CURB AFTER OVER /A /S4] (1)

HIT_ME (1)

HIT_MY (1)

HIT_POLE

@HITPOLE [SIGN AFTER GO_INTO /A /S3] (1)

@HITPOLE1 [SIGN AFTER HIT /A /S5] (1)

@HITPOLE3 [POLE AFTER GO_INTO /A /S3] (1)

@HITPOLE4 [SIGN AFTER COLLIDE_WITH /A /S5] (1)

COLLIDE_INTO_A_POLE (1)

COLLIDE_WITH_A_POLE (1)

COLLIDE_WITH_A_POWER_POLE (1)

COLLIDED_INTO_STREET_POLE (1)

COLLIDED_WITH_TWO_POLES (1)

HIT_A_HIGH_VOLTAGE_POWER_POLE (1)

HIT_A_POLE (1)

HIT_A_POWER_POLE (1)

HIT_A_TIMBER_POWER_POLE (1)

HIT_AN_ELECTRICAL_POLE (1)

HIT_POLE (1)

HIT_SIGN (1)

HIT_STREET_SIGN (1)

HIT_TIMBER_POST (1)

ROAD_AND_HIT_A_POLE (1)

STRUCK_A_POLE (1)

STRUCK_A_POWER_POLE (1)

STRUCK_A_STREET_SIGN (1)

STRUCK_A_TRAFFIC_LIGHT_POLE (1)

STRUCK_CONCRETE_POWER_POLE (1)

STRUCK_POLE (1)

HIT_TREE

@HITTREE1 [TREE AFTER VEER_INTO /A /S5] (1)

@HITTREE2 [STUMP AFTER HIT /A /S3] (1)

@HITTREE3 [TREE AFTER HIT /A /S4] (1)

@HITTREE4 [TREE AFTER COLLIDE_WITH /A /S4] (1)

ACCIDENT_VS_TREE (1)

COLLIDE_INTO_A_TREE (1)

COLLIDE_WITH_A_TREE (1)

COLLIDE_WITH_TREE (1)

COLLIDED_INTO_TREE (1)

COLLIDED_WITH_A_GROUP_OF_TREES (1)

COLLISION_WITH_A_TREE (1)

CRASH_INTO_A_TREE (1)

CRASH_INTO_TREE (1)

HIT_A_TREE (1)

HIT_SOME_TREE (1)

HIT_TREE (1)

INTO_A_TREE (1)

INTO_SOME_TREES (1)

INTO_TREE (1)

INTO_TREES (1)

SLID_INTO_A_TREE (1)

SLID_INTO_TREE (1)

STOPPED_AGAINST_A_TREE (1)

STRUCK_A_TREE (1)

STRUCK_SOME_TREES (1)

STRUCK_TREE (1)

SWIPED_A_TREE (1)

V_TREE (1)

VERSES_TREE (1)

VERSUS_TREE (1)

VS_TREE (1)

WITH_A_TREE (1)

HOOK_TURN (1)

HOUSE (1)

I_ (1)

I_HAVE (1)

I_WAS (1)

IMPACT (1)

IN_EMERGENCY_LANE (1)

IN_FRONT

@INFRONT1 [FRONT AFTER IN /A /S1] (1)

@INFRONT3 [IN BEFORE FROM BEFORE OF /A /D2/D2] (1)

IN_FROM_OF (1)

IN_FRONT (1)

INFRONT (1)

ON_FRONT (1)

IN_FRONT_OF_ME

@INFRONTOFME1 [FRONT_OF_HIM AFTER ON /A /S2] (1)

IN_FRONT_OF_CLIENT (1)

IN_FRONT_OF_HER (1)

IN_FRONT_OF_HIM (1)

IN_FRONT_OF_ME (1)

IN_FRONT_OF_PATIENT (1)

INFRONT_OF_CLIENT (1)

INFRONT_OF_HER (1)

INFRONT_OF_HIM (1)

INFRONT_OF_ME (1)

INFRONT_OF_PATIENT (1)

INTO_MY_PATH (1)

ON_FRONT_OF_HIM (1)

INTERSECTION

@CROSSING [CROSS AFTER AT /A /S2] (1)

CROSSROAD (1)

INTERSECTION (1)

TINTERSECTION (1)

INTO (1)

INTO_MY (1)

JUMP

@JUMP1 [JUMP NOT NEAR MOTOR_VEHICLE /A /S2] (1)

@JUMP2 [JUMP NOT NEAR SIDEWAY /A /S2] (1)

JUMP (1)

KANGAROO

KANGAROO (1)

ROO (1)

WALLABY (1)

LAND (1)

LANE

CARRIAGEWAY (1)

LANE (1)

LARGE (1)

LEFT (1)

LEFT_LANE

L_LANE (1)

LEFT_LANE (1)

LEFT_SIDE

LEFT_HAND_SIDE (1)

LEFT_SIDE (1)

LEFTHAND_SIDE (1)

PASSENGER_SIDE (1)

LIGHT (1)

LOST_CONTROL

@LOSTCONT [GRIP AFTER LOSE /A /S3] (1)

COULD_NOT_CONTROL (1)

COULDNT_GET_CONTROL (1)

LOOSE_CONTROL (1)

LOSE_BALANCE (1)

LOSE_CONTROL (1)

LOSE_TRACTION (1)

LOW_SPEED (1)

LUGGAGE (1)

MECHANICAL_FAILURE

@MECHFAIL1 [LOCK AFTER WHEEL /A /D3] (1)

@MECHFAIL2 [LOCK AFTER BRAKE /A /S1] (1)

@MECHFAIL3 [LOCK AFTER TYRE /A /S3] (1)

@MECHFAIL4 [BRAKE NEAR FAIL /A /S3] (1)

@MECHFAIL5 [STUCK NEAR ACCELERATOR /A /S3] (1)

@MECHFAIL6 [SMOKE BEFORE COME_OUT /A /S4] (1)

@TYRE_BLEW1 [TYRE BEFORE BLOW /A /S5] (1)

@TYRE_BLEW2 [TYRE NEAR FLAT /A /S5] (1)

ACCELERATOR_APPEAR_TO_JAM (1)

BRAKE_DONT_WORK (1)

BRAKE_FAIL (1)

BRAKE_LOCKED (1)

FAULTY (1)

GO_OUT_OF_GEAR (1)

LOCK_BIKE (1)

MOTORCYCLE_STALL (1)

REAR_TYRE_BURST (1)

SMOKE (1)

STALL (1)

TYRE_BLEW (1)

MEDIAN_STRIP

ISLAND (1)

MEDIAN_STRIP (1)

MEDIUM_STRIP (1)

NATURE_STRIP (1)

THOROUGHFARE (1)

TRAFFIC_ISLAND (1)

MEDICAL_CONDITION

APNEA (1)

CONVULSING (1)

DIZZY (1)

EPILEPTIC (1)

FAINTED (1)

SEIZURE (1)

MERGE (1)

MISS (1)

MOTOR_VEHICLE

@MV [WHEEL BEFORE DRIVE /A /S1] (1)

4WD (1)

MOTOR_VEHICLE (1)

MOTORCYCLE (1)

MOTORCYCLIST

MOTOR_CYCLIST (1)

MOTORCYCLIST (1)

MY (1)

MY_LANE (1)

MY_MOTOR_VEHICLE

@MYMV [MY BEFORE MOTOR_VEHICLE /A /S5] (1)

MY_MOTOR_VEHICLE (1)

MY_PATH (1)

MY_SIDE (1)

NOT_PERMITTED (1)

OF_TRUCK

OF_A_TRUCK (1)

OF_TRUCK (1)

OFF (1)

ON (1)

ONCOMING

INCOMING (1)

ON_COMING (1)

ONCOMING (1)

TOWARD_ME (1)

ONTO (1)

ONTO_ROAD

ON_ROAD (1)

ONTO_ROAD (1)

ONTO_STREET (1)

ONTO_THE_ROAD (1)

ONTO_THE_STREET (1)

OPPOSITE (1)

OTHER (1)

OUR (1)

OUT_OF_CONTROL (1)

OVER (1)

OVERCORRECT

OVER_CORRECT (1)

OVER_CORRECTED (1)

OVERCORRECTED (1)

OVERSHOT (1)

WIDE (1)

OVERTAKE

OVER_TAKE (1)

OVERTAKE (1)

PASS (1)

PARK (1)

PASSENGER (1)

PEDESTRIAN (1)

PEDESTRIAN_CROSS (1)

PETROL_STATION (1)

POLE

LIGHT_POLE (1)

LIGHTPOLE (1)

PILLAR (1)

POLE (1)

POST (1)

POWER_POLE (1)

POWERPOLE (1)

PYLON (1)

PULL_OUT

COME_OUT (1)

PULL_OUT (1)

PULL_OVER

@PULLOVER [PULL BEFORE GROUND /A /S5] (1)

PULL_OVER (1)

PUSH (1)

RAN (1)

RAN_INTO

RAN_INTO (1)

RIDE_INTO (1)

RAN_OVER

@RANOVER [RAN NEAR OVER /A /S5] (1)

@RANOVER1 [DRIVE BEFORE OVER /A /S5] (1)

RAN_OVER (1)

REALISE (1)

REAR (1)

REAR_END

@REAREND1 [HIT BEFORE FROM_BEHIND /A /S5] (1)

@REAREND10 [REAR AFTER HIT /A /S4] (1)

@REAREND2 [FROM_BEHIND AND HIT_ME /A /S] (1)

@REAREND3 [CLIPPED NEAR BACK_WHEEL /A /S5] (1)

@REAREND4 [BACK AFTER COLLIDE /A /S5] (1)

@REAREND5 [BACK NEAR HIT /A /S4] (1)

@REAREND6 [BACK AFTER COLLIDE_INTO /A /S5] (1)

@REAREND7 [HIT BEFORE MOTOR_VEHICLE BEFORE IN_FRONT /A /S3/D3] (1)

@REAREND8 [REAR AFTER STRUCK /A /S4] (1)

@REAREND9 [REAR NOT BEFORE IMPACT_WITH /A /S4] (1)

BEHIND_BY (1)

CLIP_THE_BACK (1)

CLIPPED_BACK_OF (1)

CLIPPED_THE_BACK (1)

CLIPPED_TO_THE_LEFT_REAR (1)

COLLIDE_INTO_REAR (1)

COLLIDE_INTO_THE_REAR (1)

COLLIDE_WITH_REAR (1)

COLLIDE_WITH_THE_REAR (1)

COLLIDED_WITH_THE_CAR_IN_FRONT (1)

COLLIDED_WITH_THE_MOTOR_VEHICLE_IN_FRONT (1)

CRASHED_INTO_THE_BACK (1)

DRIVER_REAR_END (1)

HIT_FROM_BEHIND (1)

HIT_HIM_FROM_BEHIND (1)

HIT_IN_THE_REAR (1)

HIT_ME_FROM_BEHIND (1)

HIT_MY_BACK (1)

HIT_REAR (1)

HIT_THE_BACK (1)

HIT_THE_MOTOR_VEHICLE_IN_FRONT (1)

HIT_THE_REAR (1)

HIT_THE_TRUCK_FROM_BEHIND (1)

HIT_US_FROM_BEHIND (1)

IMPACTED_IN_REAR (1)

INTO_BACK_OF (1)

INTO_THE_BACK (1)

INTO_THE_REAR (1)

NOSE_TO_TAIL (1)

PLOUGHED_INTO_THE_BACK (1)

RAN_INTO_BACK (1)

RAN_INTO_THE_BACK (1)

REAR_END (1)

REAR_END_A_STATIONARY (1)

REAR_END_ANOTHER (1)

REAR_END_BY_A_TRUCK (1)

REAR_END_BY_ANOTHER (1)

REAR_ENDED (1)

REAR_OF_ANOTHER (1)

REAREND (1)

RUN_IN_THE_BACK_OF (1)

RUNNING_IN_THE_BACK_OF (1)

RUNNING_INTO_THE_BACK (1)

SMASH_INTO_THE_REAR (1)

STRIKING_REAR (1)

STRUCK_FROM_BEHIND (1)

STRUCK_ME_FROM_BEHIND (1)

STRUCK_MY_CAR_AT_REAR (1)

STRUCK_ON_THE_LEFT_REAR (1)

STRUCK_REAR (1)

STRUCK_THE_REAR (1)

TBONED_AT_REAR (1)

RECOLLECTION

DO_NOT_KNOW (1)

RECALL (1)

RECALL_OF_EVENT

CLIENT_HAVE_NO_RECALL (1)

DO_NOT_RECALL (1)

DONT_KNOW_WHAT_HAPPENED (1)

MEMORY_OF_ACCIDENT (1)

MEMORY_OF_THE_ACCIDENT (1)

NO_IDEA_HOW_ACCIDENT_HAPPENED (1)

NOT_MEDICALLY_ABLE_TO_ADVISE (1)

NOT_SURE_WHAT (1)

PATIENT_HAVE_NO_RECALL (1)

PATIENT_UNABLE (1)

PATIENT_UNABLE_TO_EXPLAIN (1)

PATIENT_UNABLE_TO_RECALL (1)

RECALL_ACCIDENT (1)

RECALL_DESCRIPTION_AS_PER_AMBULANCE (1)

RECALL_OF_ACCIDENT (1)

RECALL_OF_EVENTS (1)

RECALL_OF_THE_ACCIDENT (1)

RECALL_OF_WHAT_HAPPENED (1)

RECALL_THE_ACCIDENT (1)

REMEMBER (1)

REMEMBER_ACCIDENT (1)

REMEMBER_ANYTHING (1)

REMEMBER_THE_ACCIDENT (1)

REMEMBER_WHAT_HAPPENED (1)

UNABLE_TO_EXPLAIN (1)

UNABLE_TO_REMEMBER (1)

UNCONSCIOUS_UNKNOWN_DETAILS (1)

RED (1)

RED_LIGHT (1)

REMOVE

OFF_LOAD (1)

REMOVE (1)

REST (1)

REVERSE

@REVERSE [BACK BEFORE INTO /A /S5] (1)

@REVERSE2 [BACK BEFORE OUT /A /S4] (1)

BACK_INTO (1)

BACK_UP (1)

REVERSE (1)

RIDGE

CREST (1)

RIDGE (1)

RISE (1)

RIDING (1)

RIGHT (1)

RIGHT_SIDE

DRIVER_DOOR (1)

DRIVER_SIDE (1)

DRIVERS_SIDE (1)

RIGHT_SIDE (1)

ROAD (1)

ROAD_CONDITIONS

@ROADCOND1 [OIL BEFORE ON BEFORE ROAD /A /S5/D5] (1)

@ROADCOND2 [SURFACE AFTER SLIPPERY /A /S3] (1)

@ROADCOND3 [ROAD AFTER SLIPPERY /A /S3] (1)

@ROADCOND4 [ROAD AFTER FUEL_ON /A /S5] (1)

@ROADCOND5 [RUT AFTER CAUGHT /A /S5] (1)

@ROADCOND6 [BAD_CONDITION NEAR ROAD /A /S5] (1)

BLACK_ICE (1)

CORRUGATION (1)

GREASY (1)

POT_HOLE (1)

POTHOLE (1)

ROAD_SURFACE (1)

RUT (1)

UNEVEN_GROUND (1)

ROAD_RAGE (1)

ROCK

BOULDER (1)

ROCK (1)

STONE (1)

STUMP (1)

ROLL

FLIP (1)

OVERTURN (1)

ROLL (1)

TIP (1)

TURN_OVER (1)

ROLL_OVER (1)

ROOF (1)

ROUNDABOUT

ENTER_THE_ROUNDABOUT (1)

ROUND_A_ABOUT (1)

ROUND_A_BOUT (1)

ROUND_ABOUT (1)

ROUNDABOUT (1)

THROUGH_ROUND (1)

SAME_DIRECTION (1)

SAW

NOTICED (1)

SAW (1)

SEVERAL_TIMES

MULTIPLE_TIME (1)

SEVERAL_TIME (1)

SIDE

EDGE (1)

SIDE (1)

SIDE_OF (1)

SIDE_OF_ROAD

SIDE_OF_HUME (1)

SIDE_OF_OF_XXXX (1)

SIDE_OF_ROAD (1)

SIDE_OF_TEH_ROAD (1)

SIDE_OF_THE_FREEWAY (1)

SIDE_OF_THE_HIGHWAY (1)

SIDE_OF_THE_HUME (1)

SIDE_OF_THE_ROAD (1)

SIDE_OF_THE_TRACK (1)

SIDE_OF_THE_XXXX (1)

SIDE_ROAD

SIDE_RD (1)

SIDE_ROAD (1)

SIDE_STREET (1)

SIDE_SWIPE

SIDE_SWIPE (1)

WIDE_SWIPE (1)

SINGLE (1)

SIT (1)

SLIDE_OVER (1)

SLIP

AQUAPLANE

AQUA_PLANE (1)

HYDROPLANE (1)

SKID (1)

SLIDE (1)

SLIP (1)

SLOW (1)

SLOW_DOWN

SLOW_DOWN (1)

SLOWDOWN (1)

SPIN_AROUND

DEGREE (1)

SPIN (1)

SPIN_AROUND (1)

SPUN_ACROSS (1)

STAND (1)

STATIONARY

HALT (1)

PAUSE (1)

STATIONARY (1)

STILL (1)

STEER (1)

STEP (1)

STOP (1)

STOP_SIGN (1)

STRAIGHT (1)

STREET (1)

STUCK

PIN_ME (1)

SANDWICH (1)

STUCK (1)

WEDGE (1)

SUBSTANCES

ALCOHOL (1)

DRUG (1)

DRUNK (1)

HEROIN (1)

INTOXICATED (1)

MARIJUANA (1)

SWERVE

FISH_TAIL (1)

FISHTAIL (1)

FISHTAILED (1)

FISHTAILING (1)

SWERVE (1)

SWERVE_TO_AVOID

SWERVE_TO_AVOID (1)

SWERVE_TO_MISS (1)

SWERVED_LEFT_TO_AVOID (1)

SWERVED_RIGHT_TO_AVOID (1)

TABLE (1)

TAKE (1)

TBONE

COLLIDE_DRIVERS_SIDE_DOOR (1)

COLLIDED_FRONT_DRIVERS_SIDE (1)

COLLIDED_WITH_FRONT_DRIVERS_SIDE (1)

COLLIDED_WITH_MY_RIGHT_HAND_SIDE_OF_VEHICLE (1)

HIT_MY_CAR_ON_MY_DRIVER_SIDE_DOOR (1)

HIT_MY_CAR_ON_THE_RIGHT_SIDE (1)

HIT_ON_HER_SIDE (1)

HIT_ON_THE_DRIVERS_SIDE (1)

IMPACTED_ON_DRIVERS_DOOR (1)

SMASHED_INTO_THE_REAR_OF_THE_DRIVERS_SIDE (1)

STRUCK_DRIVER_SIDE (1)

STRUCK_ON_DRIVER_DOOR (1)

STRUCK_ON_DRIVER_SIDE (1)

STRUCK_ON_DRIVERS_SIDEN (1)

STRUCK_ON_LEFT (1)

STRUCK_ON_THE_DRIVER_SIDE (1)

STRUCK_ON_THE_LEFT (1)

STRUCK_ON_THE_PASSENGER_SIDE (1)

STRUCK_SIDE (1)

TBONE (1)

TBONE_ON (1)

THE_FRONT (1)

THEIR (1)

THROUGH (1)

TOWING

CARAVAN (1)

TOW (1)

TRAILER (1)

TRACK (1)

TRAFFIC (1)

TRAFFIC_LIGHT

@ATLIGHTS [AT BEFORE LIGHT /A /S5] (1)

SET_OF_LIGHT (1)

TRAFFIC_LIGHT (1)

TRAIN (1)

TRAM (1)

TRAVEL (1)

TREE

BUSH (1)

FOLIAGE (1)

ROOT (1)

SCRUB (1)

TREE (1)

TRIED_TO_AVOID (1)

TRIED_TO_CORRECT (1)

TRIED_TO_STOP (1)

TURN

TUNING_INTO (1)

TURN (1)

TURN_ACROSS (1)

TURN_IN_FRONT (1)

TURN_LEFT

LEFT_HAND_TURN (1)

TURN_L (1)

TURN_LEFT (1)

TURN_MOTORVEHICLE

@TURNMV [TURN NEAR MOTOR_VEHICLE /A /S3] (1)

TURN_OUT (1)

TURN_RED (1)

TURN_RIGHT

RIGHT_HAND_TURN (1)

TURN_R (1)

TURN_RIGHT (1)

TURN_TO_MISS (1)

TYRE (1)

U_TURN

@U_TURN1 [TURN AFTER U /A /S1] (1)

U_TURN (1)

UTURN (1)

UNABLE_TO_STOP (1)

UNDER (1)

UNKNOWN

NOT_LISTED (1)

POLICE_REPORT (1)

UNKNOWN (1)

UNSURE (1)

UNOCCUPIED (1)

VAN (1)

VEER

CAREER (1)

DRIFT (1)

SWAY (1)

VEER (1)

VEER_ACROSS_LANE (1)

VEER_INTO (1)

VEER_OFF_ROAD

@VEER_OFF_ROAD [FORCE_ME BEFORE OFF_THE_ROAD /A /S6] (1)

@VEEROFFROAD [ROAD AFTER GO_OFF /A /S3] (1)

DRIVE_OFF_ROAD (1)

DROVE_OFF_ROAD (1)

GOING_OFF_THE_ROAD (1)

LEFT_THE_ROAD (1)

MOTOR_VEHICLE_VEER (1)

RAN_OFF_ROAD (1)

RUN_OFF_ROAD (1)

RUN_OFF_THE_ROAD (1)

VEER_OF_ROAD (1)

VEER_OF_THE_RD (1)

VEER_OF_THE_ROAD (1)

VEER_OFF (1)

VEER_ONTO (1)

VEHICLE (1)

VERSES

VERSES (1)

VERSUS (1)

VS (1)

VISIBILITY

@VISIB1 [SUN BEFORE EYES /A /S5] (1)

DAWN (1)

DUST (1)

FOG (1)

SUN (1)

VISIBILITY (1)

WAIT (1)

WALK (1)

WALL (1)

WAS_STOP (1)

WASHOUT

WASH_OUT (1)

WASHOUT (1)

WET_CONDITIONS

@POORWEATHER1 [WEATHER AFTER POOR /A /S3] (1)

ICE (1)

RAIN (1)

ROAD_WAS_WET (1)

SHOWER (1)

STORM (1)

WATER_ON_ROAD (1)

WATER_ON_THE_ROAD (1)

WET (1)

WET_AND_SLIPPERY (1)

WET_CONDITIONS (1)

WET_ROAD (1)

WET_TRAM_TRACK (1)

WET_WEATHER (1)

WHEEL (1)

WINDY_CONDITIONS

GUST (1)

WIND (1)

WITH_A_TRAM (1)

WOOD_LOG

BRANCH (1)

LOG (1)

WOOD (1)

WOULD_NOT (1)

WRONG (1)

WRONG_SIDE

OVER_DOUBLE_LINES (1)

WRONG_SIDE (1)
