# Supplementary material for: Classification of road traffic injury collision characteristics using text mining analysis: Implications for road injury prevention
Source: PLoS One. 2021 Jan 27;16(1):e0245636. doi: 10.1371/journal.pone.0245636 (PMC7840051; doi:10.1371/journal.pone.0245636)
Supplement: S4 File — (DOCX) [file pone.0245636.s004.docx]

**S4 File: Detailed description of the text data preparation and text analysis**

The text description of the injury event was first pre-processed in Microsoft Excel (Professional Plus, 2019), where all text was converted to lower case, and a macro was used to convert specific phrases and punctuation into a consistent term (e.g., t_bone for t-bone, tbone, t – bone etc.). Symbols were then replaced with words, and all remaining punctuation was removed.

All injury event descriptions, case identifier, road user group and fault attribution group were imported into QDA Miner version 5, and loaded into Wordstat 7.1.22 for content analysis. The language was set to English, and the corpus was checked for spelling errors which were manually corrected following review of the terms in context if the suggested correction was ambiguous. All text data were analysed at the level of the document, which comprised all text provided for each person’s injury event description.

Given the varied nature of the text data we could not analyse linguistic complexity (e.g., tense, pluralisation) or grammatical structure of the injury event descriptions (i.e., the order in which agents or operators, verbs and outcomes were given in the text narrative), unless they were recognised as a common phrase (e.g., “fell off bike”, “lost control”, “veer off road”). Most variations of keywords were lemmatized to third-person, singular and past tense for analysis, unless another form was contextually more appropriate.

While generating the categorisation dictionary, stop words were applied and tailored to the study text, adapting the Provalis **exclusion list** for English terms throughout all phases of the text mining analysis, consistent with previous studies (Brooks, 2008); see Supplementary Materials 1. The exclusion list defined common less informative grammatical components (e.g., it, he, the, a) and other ambiguous terms that did not contribute meaningfully to the text analyses. Stop words were added to the exclusion list if they had a very high frequency and were considered unlikely to help classify unique crash circumstances or to differentiate between fault groups. High frequency terms were added to the exclusion list and were omitted from exploratory cluster analyses.

The English **lemmatization dictionary** provided by Provalis was tailored to this dataset, and included stemming rules (e.g., *accelerate, accelerated* and *accelerating* were all analysed as *accelerated*), and consolidation of some similar terms. This enabled the subsequent analysis of a single common, relevant or base term, and aimed to reduce the variability in the dimensions analysed (Supplementary Materials 2). A **categorisation dictionary** was developed through multiple cycles of analyses, including cluster analyses and reviewing keywords in context. Keywords with a frequency of <10 were added to the categorization dictionary where possible to ensure that they were included in subsequent cluster analyses and injury event classification (Supplementary Materials 3). Common phrases were included in the categorisation dictionaries, and were restricted to ordered keyword occurrences comprising between two and five terms with a minimum frequency of 5 occurrences across the corpus. All phrases first identified by Wordstat were added to a categorization dictionary and reviewed in context for relevance to the categorization dictionary. The phrases were reviewed in context for meaning and expression to identify and correct any erroneous substitutions in the lemmatization rules that had altered or diminished the meaning of the expression (e.g., initial substitution of asleep with fatigue was dropped as “fall asleep at the wheel” was more appropriate than “fall fatigue at the wheel”). Finally, after reviewing words in context and attempting to classify text descriptions into crash classifications, combinations of some terms were included in the categorisation dictionary using proximity and ordering rules (e.g., “brake” within one word before or after “suddenly” = “brake suddenly”; “cut” within three words before “off” = “cut off”).

The lemmatization and categorization dictionaries were discussed among the authors and refined before undertaking exploratory analysis of crash classifications. The only large body of terms consolidated in the lemmatization dictionary rather than categorisation dictionaries were terms used to refer to a motor vehicle given that 29 terms were found to refer to motor vehicles (i.e., specific vehicle types or makes). This then simplified the classification of phrases at later stages of analysis and data extraction. Simple revisions to the exclusion list, lemmatization dictionary and categorization dictionary were made within Wordstat. Extensive revisions were more efficiently applied in an external text editing program and uploaded to Wordstat.

Multiple iterations of **exploratory cluster analyses** for each road user group were used to examine the co-occurrence of phrases and keywords in the corpus to further refine the exclusion list, and lemmatization and categorization dictionaries. The exploratory cluster analyses were restricted to terms that had a frequency >=5 occurrences, with a maximum of 150 terms based on the TF*IDF, and omission of any items that occurred in more than 50% of cases. The TF*IDF indicates the relative weight of words within the corpus, whereby the frequency of terms is weighted by inverse document frequency. Higher TF*IDF therefore indicates both a higher frequency of the term across the corpus, but can also reduce the discrimination of terms across documents. The relationship between terms was restricted to those that occurred within 5 words of each other, and the level of categorisation was set to the highest level in the dictionary, classifying all “child” terms at the highest level of the category name. Cluster dendograms were reviewed iteratively, and keywords in each cluster were reviewed in context in order to identify the combinations of terms that were indicative of unique types of crash classifications. These exploratory analyses were undertaken separately for each road user group. Upon reviewing the exploratory cluster analysis results several keywords or phrases were again re-categorised, and many phrases were omitted, reduced to the simplest component of the phrase, or combined within a parent category to ensure that terms were categorised as concisely as possible (e.g., there were 12 phrases that described driving off the road, and 49 phrases that described a rear-end collision).

The case occurrence of terms from the categorisation dictionary were exported to Stata, Version 15, for linkage and analysis with other participant characteristics via the case identifier. Crash classifications were classified in Stata using combinations of the exported keywords based on iterative review of crash descriptions from the clusters. Where possible the type of crash scenario was coded according to the VicRoads Definitions for Classifying Accidents (DCA) (Vic Roads, 2013), which classify collision and near-collision events from the perspective of the driver of a vehicle. As the level of detail provided in the majority of injury event descriptions was not sufficient to apply a specific DCA code, the range of codes for broader categories of crash classifications were applied. The main DCA categories included: pedestrian impacts (DCA 100-108); turning vehicles from an adjacent direction (DCA 110-118) or from opposing direction (DCA 121 or 125); head on collision (DCA 120); collisions in the same direction (i.e., rear-end collisions; DCA 130-137); manoeuvring (e.g., u turns, leaving or entering a car park; DCA 140-148); overtaking or changing lanes (DCA 150-154); collision with an animal (DCA 167); events in which the vehicle lost control on a straight path (DCA 170-175), or on a curve (DCA 180-184); and other miscellaneous events involving falls from a vehicle, being struck by a load falling from a vehicle, hitting a train/tram/railway infrastructure, or being hit by a runaway parked car (DCA 190-194). Injury events could be classified into more than one scenario. Unfortunately, administrative DCA coding for each injury event was not available in this study for validation of the text mining results; however, to optimise accuracy all crash classifications were cross referenced with the free text.

## References

Brooks, B., 2008. Shifting the focus of strategic occupational injury prevention: Mining free-text, workers compensation claims data. Safety Science 46, 1-21.

Vic Roads, 2013. Appendix C: Definitions for Classifying Accidents (DCA) Chart, Crashstats user guide: Road Crash Statistics. Vic Roads, Victoria.
