# Supplementary material for: Classification of road traffic injury collision characteristics using text mining analysis: Implications for road injury prevention
Source: PLoS One. 2021 Jan 27;16(1):e0245636. doi: 10.1371/journal.pone.0245636 (PMC7840051; doi:10.1371/journal.pone.0245636)
Supplement: Attachment — Submitted filename: 1a. Response to Reviewers.docx [file pone.0245636.s005.docx]

Dear Editor,

We respond below, point by point, to the suggestions made by the reviewers and indicate where changes have been made in the marked up copy of the revision.

We have also made the following changes, as requested by the editorial team:

- Included an ethics statement on Page 4
- Uploading the supplementary materials as separate documents.
- Correction to the reference to Table 6 on Page 19 which had originally been included in the manuscript as Table 2.

On behalf of my coauthors,

Dr Melita Giummarra

**Reviewer #1: This paper investigates road traffic injury collision characteristics in Victoria, Australia. The investigation is based on the data from Victorian State Trauma Registry using text mining techniques, which provide many detailed and structured information on occupants involved in the reported crashes. The research topic is interesting and worth of investigation.**

**While the occupant-specific factors are enriched significantly, developing statistical regression models (such as logit/probit model) would provide a** **deeper insight on the effects of these factors (as well as those specific to vehicle, roadway, and environment, etc.) on crash injury severity, which is consistent with many previous studies on traffic safety analysis. Given the length of this article, it can be a direction for future research.**

**RESPONSE**: This is a very good suggestion. In the initial draft of the paper we had included multinomial logistic regression to determine which characteristics were associated with being at fault for each of the road user groups. A team decision was made, however, to omit this from the paper as the data presented from the text mining and descriptive statistics were already quite complex and detailed.

We have taken this comment on board in our revision, however, and have included a suggestion on Page 37 for future research to “extend the methods used in the present study using predictive modelling in order to generate deeper insights into the association between fault attribution and/or traffic collision characteristics (e.g., single vehicle versus multiple vehicles) and circumstances (e.g., environmental conditions, driver characteristics) on a range of factors, including crash severity, injury severity, survival rates and long-term outcomes.”

**Besides, driver at fault or not is a key factor considered in the empirical analysis.**

**RESPONSE**: That is correct. Unfortunately, the fault-related data recorded in the compensation claim lodgment only relates to whether another party was at fault. This is because people whose injuries occur when another is partially or fully at fault may be entitled to additional compensation through a common law claim. The compensation scheme does not typically need to know whether the injured person was also at fault. They only need to know whether they were reckless/negligent (e.g., due to intoxication) or intentionally caused the collision and were convicted of culpable offences, which would limit their entitlements. We did not have access to those data through the compensation claim records

**More existing findings on its safety effect can be referred to support the results in this research. For example, the following article analyzed the effects of driver at fault on the injury severities of the own and other driver-vehicle units in two-vehicle crashes: The interactive effect on injury severity of driver-vehicle units in two-vehicle crashes. Journal of Safety Research, 2016, 59: 105-111.**

**RESPONSE**: Thank you for this suggestion. We have now referred to that study on Page 35, to highlight the findings that were consistent with the two Australian studies examining fault attribution.

**Reviewer #2. This paper characterised crash characteristics of road traffic collisions in Victoria, Australia, and examined the relationship between crash characteristics and fault attribution. The topic is interesting. The methods sound. The results are meaningful and useful. It is suggested to be published.**

**RESPONSE**: We thank Reviewer 2 for their positive comments on our manuscript.
